# A Framework for an Effective Healthy Longevity Clinic

**DOI:** 10.14336/AD.2024.0328-1

**Published:** 2025-07-15

**Authors:** Sergey Mironov, Olga Borysova, Ivan Morgunov, Zhongjun Zhou, Alexey Moskalev

**Affiliations:** ^1^Longaevus Technologies LTD, London, United Kingdom.; ^2^Human and health division, DEKRA Automobil GmbH, Chemnitz, Germany.; ^3^School of Biomedical Sciences, University of Hong Kong, Hong Kong.; ^4^Institute of biogerontology, National Research Lobachevsky State University of Nizhni Novgorod (Lobachevsky University), Nizhny Novgorod, Russia.; ^5^Gerontological Research and Clinical Center, Russian National Research Medical University, Moscow, Russia.

**Keywords:** healthy longevity, longevity medicine, aging biomarkers, clinical management

## Abstract

In the context of an aging global population and the imperative for innovative healthcare solutions, the concept of longevity clinics emerges as a timely and vital area of exploration. Unlike traditional medical facilities, longevity clinics offer a unique approach to preclinical prevention, focusing on "prevention of prevention" through the utilization of aging clocks and biomarkers from healthy individuals. This article presents a comprehensive overview of longevity clinics, encompassing descriptions of existing models, the development of a proposed framework, and insights into biomarkers, wearable devices, and therapeutic interventions. Additionally, economic justifications for investing in longevity clinics are examined, highlighting the significant growth potential of the global biotechnology market and its alignment with the goals of achieving active longevity. Anchored by an Analytical Center, the proposed framework underscores the importance of data-driven decision-making and innovation in promoting prolonged and enhanced human life. At present, there is no universally accepted standard model for longevity clinics. This absence highlights the need for additional research and ongoing improvements in this field. Through a synthesis of scientific research and practical considerations, this article aims to stimulate further discussion and innovation in the field of longevity clinics, ultimately contributing to the advancement of healthcare practices aimed at extending and enhancing human life.

## INTRODUCTION

In today's rapidly advancing world, the need to develop a robust concept for longevity clinics has become increasingly pressing [1, 2]. Unlike wellness centers or traditional medical facilities, longevity clinics represent a unique approach to preclinical stage prevention, aptly termed "prevention of prevention." While primary prevention focuses on biomarkers associated with disease, longevity clinics utilize aging clocks based on data from practically healthy individuals. This innovative approach allows for the assessment and monitoring of health status with an emphasis on age groups rather than disease presence, enabling more precise and early interventions.

Given the relevance of this topic, we have decided to develop a framework for longevity clinics that addresses four key areas:

Description of existing models of longevity clinics and their proposed interventions.

Development of a framework for a longevity clinic, including its structure, data processing algorithms, and analytical report models.

Summary of the current knowledge on biomarkers, wearable devices, and therapies used in this field.

Economic justification and evaluation of the investment potential of longevity clinics.

Furthermore, demographic trends revealing an aging population worldwide underscores the imperative for developing longevity clinics. According to recent statistics from WHO by 2050, the world’s population of people aged 60 years and older will double (2.1 billion). The number of persons aged 80 years or older is expected to triple between 2020 and 2050 to reach 426 million (www.who.int/news-room/fact-sheets/detail/ageing-and-health). This demographic shift emphasizes the urgent need for innovative healthcare solutions that cater to an aging population's specific needs and challenges [2-4].

Biotechnologies are continuously and rapidly evolving, with one of their primary goals being the achievement of active longevity. On the other hand, practical healthcare is characterized by its conservatism, relying on technologies with proven effectiveness. The aim is to develop a framework of a healthy longevity clinic that leverages the latest biotechnologies to achieve practical goals, specifically, patient longevity.

The rapid development of biological sciences allows us to talk about serious achievements in the field of longevity. Unfortunately, clinical medicine often lags in translating cutting-edge technologies into practice. To bridge this gap, a new model of a longevity clinic is emerging, designed to integrate scientific discoveries into diagnostic and treatment processes to maximize active longevity. The proposed framework for a longevity clinic is based on extensive research conducted by a team of scientists and professionals with international experience in biotechnology, clinical and preventive medicine, hospital management, and financial management.

We've given careful consideration to the economic and investment aspects of the proposed longevity clinic model, particularly in light of the current global economic downturn, which has prompted investors to exercise caution and favour dynamically evolving sectors like biotechnology and longevity. In 2022, the global biotechnology market soared to USD 1023.8 billion, and projections indicate a robust 14% compound annual growth rate (CAGR) from 2023 to 2032, with an anticipated value of USD 3,672.9 billion by 2032. Key areas in biotechnology, including artificial intelligence, gene editing, tissue engineering, stem cells, real-world evidence trials, and innovative financial and management technologies, are experiencing dynamic growth in 2024. Against this backdrop, our objective is to craft a framework for a vibrant longevity clinic that harnesses cutting-edge biotechnologies to achieve tangible goals, notably enhancing patient longevity.

## MATERIALS AND METHODS

### Core concepts

The proposed framework for the longevity clinic was developed using basic concepts:

### Aging biomarkers

Biomarkers of aging are biological parameters that can predict functional capacity at a later age better than chronological age. These biomarkers aim to provide a more accurate measure of an individual's "biological age," which may differ from their chronological age. They are used to assess age-related changes, track the physiological aging process, and predict the transition into pathological states.

### Scientific Information Analytical Center (Analytical Center)

The Analytical Center is a cornerstone of the longevity clinic, managing scientific research, implementing findings in therapeutic and diagnostic processes, and aligning strategies with bioscience trends. It comprehensively acquires, processes, analyzes, and reports patient data for personalized healthcare delivery, utilizing advanced technologies like wearable devices and diagnostic tools. Through sophisticated algorithms, it generates insights for patient stratification, predictive analytics, and clinical decision support while fostering continuous improvement, research, and patient-provider engagement within the clinic.

### Comprehensive Health Profile of a Patient

A comprehensive set of data derived from a secure database, built on top of all collected data, presented as tables and/or visualizations, and regularly updated slides, providing insights into a patient's health status.

### Medical Visualization System

A Medical Visualization System is a robust tool designed to graphically present and analyze medical information for individual patients, enhancing healthcare decision-making and patient outcomes. The system integrates data from Electronic Health Records (EHRs), Laboratory Information Systems (LIS), Medical Imaging Systems (e.g., PACS), and wearable devices. The Data Integration Layer extracts, transforms, and loads (ETL) data into a centralized repository, ensuring quality and consistency through data connectors, transformation engines, and validation modules. This data is stored in relational (e.g., MySQL, PostgreSQL) or NoSQL (e.g., MongoDB, Cassandra) databases, depending on the data's scale and nature.

The Data Analysis Engine performs advanced analytics, including statistical analysis, machine learning for predictive modeling, and data mining for hidden insights. The Visualization Engine creates interactive visualizations with charts, dashboards, geospatial maps, and 3D renderings for medical imaging. Users interact with these visualizations via a web-based interface, mobile app, and collaboration tools, while the Security and Privacy Layer ensures data confidentiality, integrity, and availability through authentication, encryption, and audit logging.

The system's data flow involves collecting data from various sources, performing ETL processes, storing standardized data in the repository, conducting analytics, generating visualizations, and allowing user access through the interface. This architecture supports data-driven decision-making, identifies patterns and trends, and ultimately improves patient care and outcomes.

### Medical Database

A collection of medical data from various patients, organized for efficient search and analysis of individual patient data and parameter values across different patients.

### Data Sources and Selection Criteria

The project's development relied on three primary information sources:

Authors' work experience in organizing four preventive clinics in Europe between 2014 and 2023.

Analysis of best practices from clinics and research centers addressing longevity issues, including existing longevity clinics.

### Literature search

The selection criteria for clinics included in the best practices analysis were:

Prioritization of preventive care using both classical and modern technologies.

Commercial orientation with management focused on financial results and effectiveness.

Data openness, allowing sufficient information for analysis, typically achieved through personal involvement in clinic organization.

Our own experience has become the main source of information. The authors have experience in organizing 4 preventive clinics in the period from 2014 to 2023 in Europe. According to the terms of agreements with customers, the names of clinics cannot be disclosed, but general characteristics are given in Supplementary Table 1.

All clinics shared common features of outpatient treatment and location in ecologically clean resort areas. Longevity medicine specialists worked closely with general medicine doctors. The authors interacted directly with patients, evaluated treatment results individually and at the sample level, and were responsible for the clinics' financial results.

### Data Processing Algorithms and Analytical Report Models

The proposed framework for the longevity clinic will utilize advanced data processing algorithms and analytical report models to extract actionable insights from patient data and enhance personalized care strategies.

Data processing algorithms serve to analyze patient data and generate insights, and our model includes the following:

Clustering algorithms (e.g., K-means, hierarchical clustering) to group patients with similar health profiles or identify patterns in patient data.

Classification algorithms (e.g., decision trees, support vector machines) to predict patient outcomes or categorize patients based on specific health attributes.

Time series analysis (e.g., ARIMA, exponential smoothing) to monitor and forecast patient health trends over time.

Association rule mining to uncover relationships between patient characteristics, treatments, and outcomes.

Anomaly detection algorithms (e.g., isolation forests, local outlier factor) to identify unusual patient cases or potential health risks.

The proposed frameworkl for the longevity clinic employs various 2D models for analytical reporting. Heatmaps visualize correlations between patient attributes, treatments, and outcomes to identify patterns. Sankey diagrams illustrate patient flows through different stages of care, revealing bottlenecks or areas for improvement. Radar charts compare multiple patient attributes or outcomes simultaneously, highlighting strengths and weaknesses. Funnel plots visualize the performance of different treatments or interventions, identifying outliers or areas of exceptional performance. This model aids in enhancing patient care and optimizing treatment strategies.

These analytical report models are generated using the data processing algorithms and are designed to provide clear, actionable insights for clinicians and decision-makers.

## RESULTS

### Existing models of Longevity clinics

There is no global standard for the construction of longevity clinics. In our study, we began by identifying the limitations in the current landscape and examining existing models of longevity clinics and their diagnostic approaches and proposed interventions (Supplementary Table 2). The Discussion section delves into these limitations in detail.

Our analysis of existing longevity clinics revealed several key features essential for optimal functioning:

Objective: Longevity clinics aim to enhance active patient longevity through a blend of cutting-edge and established medical technologies.

Ethical Standards: Adherence to ethical guidelines is paramount for longevity clinics, ensuring patient welfare and regulatory compliance.

Regulatory Compliance: Longevity clinics must comply with the healthcare regulations of their respective countries and function as fully accredited healthcare institutions.

Global Collaboration: Given the international scope of biomedical science, longevity clinics should embrace global cooperation, research partnerships, and knowledge exchange.

Technological Innovation: Incorporating advanced technologies, such as big data analytics and artificial intelligence, is vital for informed decision-making and efficient operations [5, 6].

Financial Viability: As commercial ventures, longevity clinics must balance medical, scientific, and financial considerations to deliver quality care sustainably.

Based on these insights, we have developed a frameworkof a longevity clinic, including the following elements: 1) structure of the clinic; 2) analytical center estimation, 3) personnel policy; and 4) medical technologies. We analyze them sequentially.

**Figure 1.**
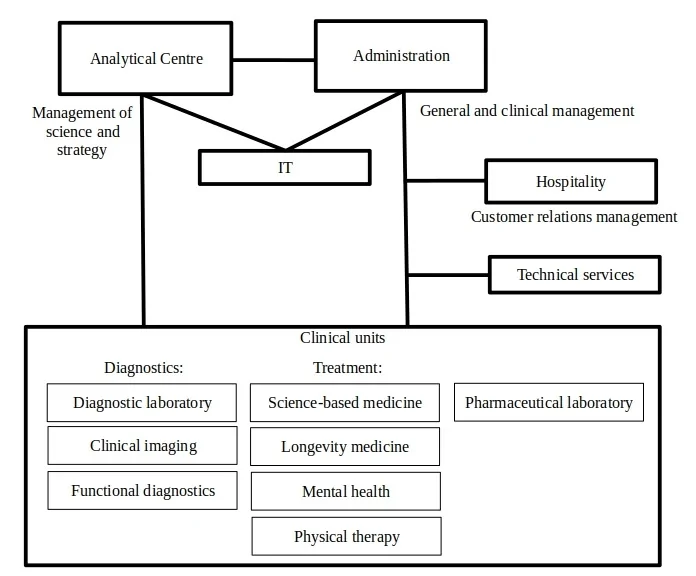
**The structure (architecture) of the longevity clinic**. This organizational structure demonstrates how an Analytical Center can be set up to efficiently manage and coordinate a range of diagnostic and treatment services by having a centralized administration providing shared resources and oversight to specialized clinical units focusing on specific aspects of healthcare delivery and medical research.

### The Structure of the Proposed Longevity Clinic Framework

The structure of the longevity clinic should be versatile and adaptable to keep pace with rapidly developing longevity technologies. By the time the clinic opens, many of the technologies known at the time of its planning will be outdated, with new ones taking their place. Therefore, the clinic should avoid crystallizing specific technologies in its structure and remain open to new advancements. This adaptability underscores the importance of a scientific center as the core of the longevity clinic. Departments engaged in practical work with patients should be organized according to the cluster principle, allowing for changes in their functions as technologies evolve. Our vision of this structure is presented in Figure 1.

Our comprehensive model of the longevity clinic is structured around several key components aimed at delivering care and innovative treatments. At its core lies the Analytical Center, a pivotal feature that drives research, innovation, and technological advancements. This center serves as the “brain” of the clinic, facilitating data analysis, research initiatives, and the integration of cutting-edge technologies. The Analytical Center, which is pivotal for research, innovation, and technological advancements, and its function in detail are described below.

Clinical units within the longevity clinic are organized to provide specialized services and diagnostic capabilities and consist of Diagnostic Units, Diagnostic Laboratories, and Treatment Units. The Diagnostics Units are particularly extensive and well-equipped, offering a wide range of tests and assessments. Many patients utilize these diagnostic services exclusively, either for routine health monitoring or to detect potential health issues. These units also play a crucial role in research activities, collecting valuable data and contributing to clinical trials.

The Diagnostic Laboratory within the clinic conducts a variety of tests, including routine screenings, metabolic analyses, genetic testing, and early cancer diagnostics. Clinical imaging services encompass advanced techniques such as MRI, sonography, and non-invasive endoscopy, while functional diagnostics include ECG, ABI, pulse variability monitoring, and EEG.

Treatment units focus on identifying risk factors for chronic diseases and providing early diagnoses of organ dysfunctions. These units offer a range of medical specialties, including science-based medicine, preventive medicine, longevity medicine, mental health services, and physical therapy. Under the umbrella of science-based medicine, the clinic offers specialized services in internal medicine, cardiology, pulmonology, gastroenterology, nephrology, endocrinology, neurology, ENT, gynecology, dermatology, and sports medicine. These specialties encompass a wide range of medical expertise, allowing for comprehensive assessments and targeted treatments tailored to individual patient needs. In addition to conventional medical practices, the clinic emphasizes modern preventive medicine, focusing on anti-aging strategies and threpsology to optimize patient health and vitality. Occupational medicine and environmental medicine are also integral components of the clinic's preventive approach, aiming to identify potential correlations between patient health indicators and environmental factors.

Longevity medicine represents a forward-looking aspect of the clinic's services, prioritizing the prevention of health risks before they manifest at clinical stages. This proactive approach includes longitudinal measurement of the aging clock and interventions aimed at mitigating age-related health decline.

Mental health services and physical therapy round out the treatment offerings, providing essential support for patients' psychological well-being and physical rehabilitation. Additionally, the clinic features a pharmaceutical laboratory dedicated to the individualized manufacture of biologically active substances, ensuring tailored treatment regimens aligned with each patient's unique requirements.

Additionally, the clinic features a pharmaceutical laboratory for the individualized manufacture of biologically active substances tailored to each patient's needs. The variability in the speed of aging among different organs underscores the necessity of considering individualized diagnostic and treatment approaches to effectively address the complexities of aging-related diseases [7].

Support services, including general management, IT support, hospitality and customer relations management, and technical services, ensure the smooth operation of the clinic. The clinic's flexibility of structure, coupled with its focus on integrating clinical services around the Analytical Center, distinguishes it from traditional healthcare models. This three-level routing of patients, combined with a scientific core, underscores the clinic's commitment to delivering personalized care and driving advancements in longevity medicine.

#### Three-Level Patient Routing System

**Figure 2.**
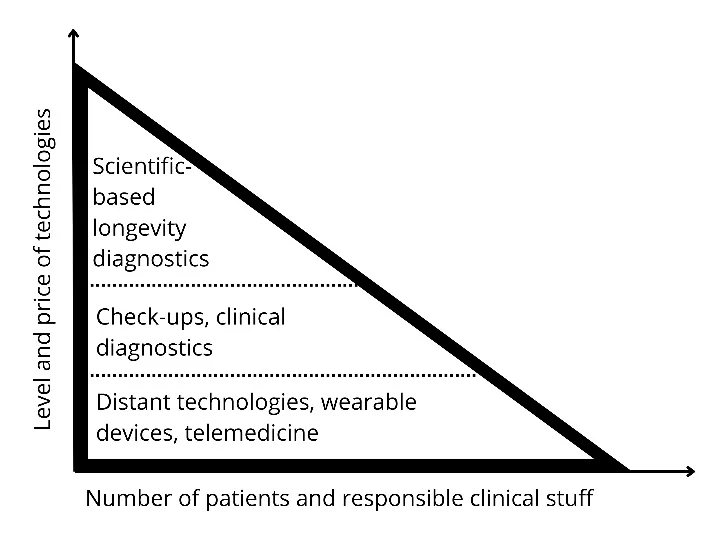
**Conceptual levels model of diagnostics in the longevity clinic.** The diagram visualizes the inverse relationship between the level/price of medical technologies and the scale at which they can be deployed in terms of patients served and staff required.

The longevity clinic stands apart from other well-known clinic models not only through its advanced technologies but also through its innovative and flexible approach to integrating new effective technologies and phasing out outdated or unproven ones. The longevity clinic is organized flexibly in terms of introducing new effective technologies and eliminating outdated or unproven technologies. Our concept includes a three-level patient routing system to ensure comprehensive care and continuous innovation (Fig. 2).

Level 1: Remote Monitoring and Telemedicine. At this level, the clinic utilizes wearable devices like smartwatches, fitness trackers, and biosensors to continuously monitor vital signs, activity levels, sleep patterns, and other health metrics. Artificial intelligence algorithms analyze the large volumes of data collected from these devices to identify potential health issues or trends. Patients have access to a user-friendly mobile app that displays their health data, offers personalized recommendations, and facilitates communication with the clinic's healthcare team. Regular telemedicine consultations via video conferencing allow for discussion of the patient's progress, addressing concerns, and adjusting treatment plans as needed. The clinic implements a "clinic at home" concept, enabling patients to perform basic diagnostic tests (e.g., blood pressure, glucose levels) using at-home devices that automatically sync data with the clinic's system. The most used wearable gadgets for self-testing health parameters are listed in Supplementary Table 3. This stage involves 24/7 patient contact worldwide, leveraging continuous monitoring and two-way communication [8]. The focus here is on the continuity and volume of research rather than depth, making medicine more accessible and convenient for patients. Those requiring in-person examinations or advanced diagnostics are directed to Level 2.

Level 2: In-Clinic Diagnostics and Treatment. This level offers comprehensive health assessments using advanced diagnostic technologies, such as whole-body MRI scans, genetic testing, and biomarker analysis. Personalized treatment plans are developed based on the patient's unique genetic profile, lifestyle factors, and health goals. The clinic incorporates cutting-edge therapies, such as stem cell treatments, gene therapies, and personalized pharmaceuticals, as they become available and proven effective. Collaborations with leading research institutions and industry partners ensure that the clinic remains at the forefront of longevity medicine, providing patients access to the latest evidence-based interventions. This stage is carried out directly in the clinic, based on the best global practices and standards, and the clinic’s own scientific developments.

Level 3: Individualized Scientific Studies. The clinic conducts in-depth scientific studies for patients with complex or rare conditions to better understand the underlying mechanisms and develop tailored treatment approaches. Advanced technologies, such as AI-driven data analysis, multi-omics profiling, and patient-specific disease models (e.g., organoids, digital twins), are utilized to gain unprecedented insights into the patient's unique biology. The clinic collaborates with a global network of experts across various disciplines (e.g., genetics, bioinformatics, systems biology) to leverage collective knowledge and accelerate discoveries. Findings from individualized studies are published in peer-reviewed journals, contributing to the growing body of knowledge in longevity medicine and potentially benefiting other patients facing similar challenges. This level represents the highest stage of work, the most technologically advanced and exclusive.

By implementing this three-level approach, the longevity clinic can provide a continuum of care that ranges from continuous remote monitoring and early intervention to highly personalized, cutting-edge treatments for complex cases. The flexible and adaptable nature of the clinic's structure allows for the rapid integration of new technologies and the elimination of outdated or ineffective ones, ensuring that patients always have access to the most promising longevity interventions.

#### Structure and Functions of the Analytical Center in the Proposed Framework of Longevity Clinic

**Figure 3.**
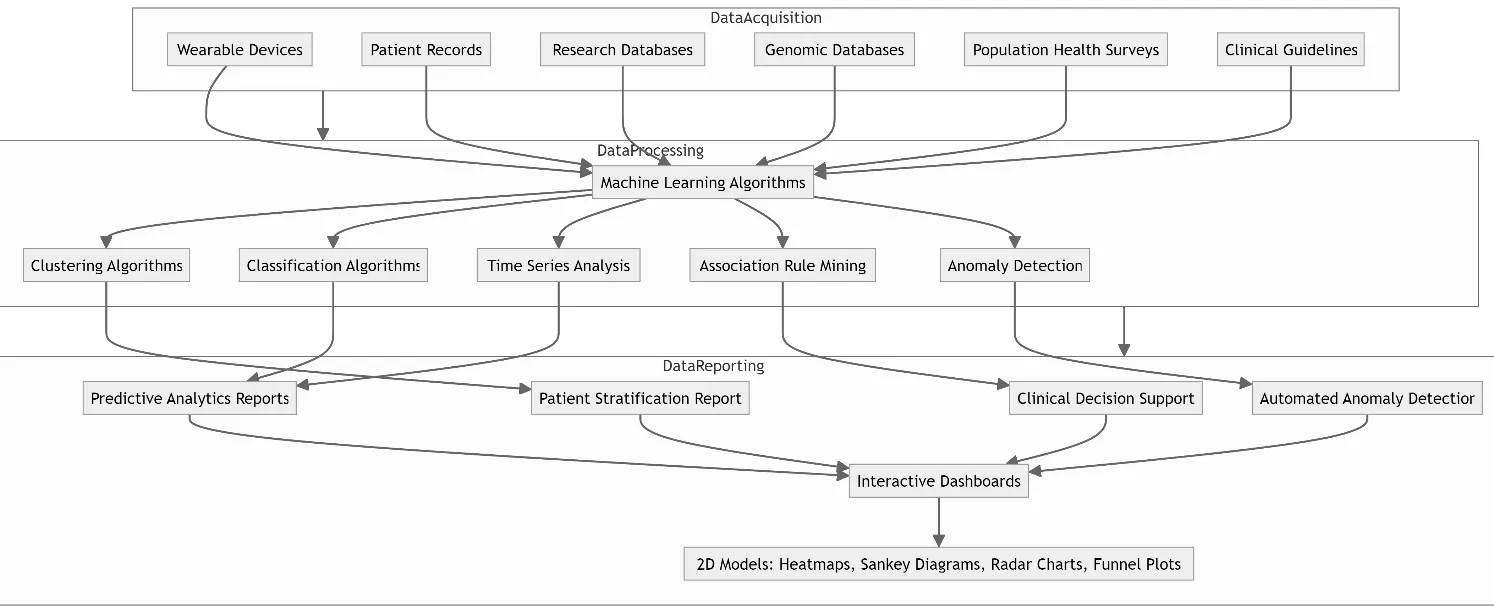
**Analytical Center Data Flow and Analytics Workflow**. The Analytical Center of the longevity clinic is crucial for personalized patient care, utilizing advanced technologies and machine learning algorithms to acquire, process, and analyze health data from wearable devices and diagnostic tools. This system generates reports for patient stratification, predictive analytics, clinical decision support, and anomaly detection, enabling proactive and personalized healthcare delivery.

The Analytical Center is the fundamental component of a longevity clinic, providing data obtaining, critical data processing, analysis, and reporting capabilities to support personalized patient care. Additionally, it plays a pivotal role in preparing educational literature for both healthcare professionals and patients, managing a centralized database for efficient information retrieval, facilitating decision support and interpretation, fostering continuous improvement and research, and promoting patient-provider engagement to ensure collaborative and informed healthcare decisions (Fig. 3).

Data obtaining. Patient data is collected through a combination of wearable devices and on-site diagnostics. Wearable devices play a pivotal role in continuously monitoring various aspects of patient health and quality of life. Our proposed longevity clinic model offers a range of devices, including multi-channel EEG bands, fitness trackers, continuous glucose monitors, ketone meters, and 24-hour ambulatory blood pressure monitors, enabling comprehensive data collection and analysis.

In our proposed framework for a longevity clinic, the acquisition of datasets for our algorithms is paramount to ensuring comprehensive and effective patient care. Leveraging diverse sources, such as patient records, research databases, wearable devices, genomic databases, population health surveys, and clinical guidelines and literature, our Analytical Center aims to develop sophisticated algorithms tailored to individual patient profiles. Surveillance systems can be used to integrate multiple data sources [9].

Data processing. Analytical Center, as a part of the proposed frameworkfor the longevity clinic, will utilize advanced data processing and machine learning algorithms to extract actionable insights from patient data, generate analythical report models and enhance personalized care strategies.

To analyze patient data and generate insights, our model employs several algorithms, including clustering algorithms like K-means, hierarchical clustering, and phenotype clustering. These algorithms group patients with similar health profiles or identify patterns in patient data and help to stratify patients based on shared characteristics, enabling targeted interventions and care strategies [10-13]. For example, K-means can segment patients based on their metabolic profiles, while hierarchical clustering can create dendrograms illustrating relationships among different patient groups.

Classification algorithms such as decision trees and support vector machines (SVMs) predict patient outcomes or categorize patients based on specific health attributes. Decision trees might be used to determine the likelihood of a patient developing cardiovascular disease [14], while SVMs can classify patients into different risk categories, such as diabetes [15]. Time series analysis methods, including ARIMA and exponential smoothing, monitor and forecast patient health trends over time, e.g. ARIMA can predict future blood pressure trends based on historical data [16, 17], while exponential smoothing can track changes in a patient's weight. Association rule mining techniques uncover relationships between patient characteristics, treatments, and outcomes and can reveal associations between specific dietary habits and improvements in biomarkers, e.g. cholesterol level [18].

Additionally, traditionally anomaly detection algorithms like isolation forests and local outlier factor, as well as the recently developed isolation-based outlying scoring measure SiNNE, will help identify unusual patient cases or potential health risks [19].

Data reporting. Based on those algorithms Analytical Center will generate the following:

Patient stratification reports grouping patients by common attributes for targeted interventions.

Predictive analytics reports forecasting individual patient trajectories and population-level trends.

Clinical decision support providing personalized treatment recommendations based on similar patient outcomes.

Automated detection of anomalies in patient data triggering alerts for potential issues needing attention

Interactive dashboards visualizing patient clusters, risk profiles, care gaps, and key performance metrics.

The analytical reports generated by these algorithms utilize various 2D models, such as heatmaps to visualize correlations between patient attributes, treatments, and outcomes; Sankey diagrams to illustrate patient flows through different stages of care; radar charts to compare multiple patient attributes or outcomes simultaneously, e.g. to display a patient's physical activity, diet quality, sleep patterns, and stress levels, helping identify areas needing attention; and funnel plots to visualize the performance of different treatments or interventions. These models provide clear, actionable insights for clinicians and decision-makers.

Integration of AI in the Analytical Center: The incorporation of artificial intelligence (AI) into our Analytical Center presents a transformative opportunity to revolutionize patient care within the longevity clinic. By harnessing AI-powered algorithms for data processing and analysis, our Analytical Center can efficiently analyze vast datasets to uncover nuanced patterns and insights, enhancing our understanding of patient health and treatment outcomes. AI-driven analytics enable longevity clinics to deeply phenotype each patient's unique aging process, identify the key levers to target, and continuously personalize interventions over time. As AI systems become more sophisticated, they may be able to replace some of the manual testing and integrate data from multiple clinics, greatly increasing the predictive power and scalability of longevity medicine [20]. However, expert physician oversight remains crucial to ensure responsible use of AI recommendations in patient care [21].

Functions of Analytical Center: The primary function of the Analytical Center is to generate comprehensive analytical reports leveraging all available patient data, facilitated by robust data processing and machine learning algorithms. These reports serve as invaluable tools for healthcare professionals, providing deep insights and actionable recommendations to optimize patient care and treatment strategies [22]. Through meticulous data analysis and algorithmic methodologies, the Center empowers clinicians with evidence-based insights, ultimately enhancing diagnostic accuracy, treatment efficacy, and patient outcomes.

Creation of a comprehensive health profile of a patient, which offers a concise, visually accessible compilation of the patient's medical history, current condition, ongoing progress, needs, lifestyle, and treatment response. It integrates analytical reports data, biochemical, genetic, and functional results from various assessments and employs dynamic formats like graphs, CT scans, MRI images, ultrasound imaging, and 3D visualizations for enhanced comprehension. It is presented with the help of the Medical Visualization System in dynamic formats and are periodically updated.

Provision of Information Technology Support for Medical Staff. The Analytical Center is staffed by specialists proficient in handling medical information systems, scripting programming languages, data mining, spreadsheets, 3D visualizations, and remote communications. These experts assist doctors with documentation, medical data processing and visualization, remote support, health coaching, and videoconferencing for second opinions.

Collaborative decision-making for patient treatment optimization. In our proposed framework, every specialist communicates their findings to the chief physician or curator, leading to a collective decision on treatment strategies. This process integrates objective and subjective data, along with predictions from algorithm models, with proper validation [23], to tailor treatments and adjust strategies based on patient responses. Emphasis is placed on risk assessment and disease prevention, with consideration given to biomarkers of aging [24-26], functional status, and lifestyle factors. Data from various sources, including fitness trackers, bioimpedance imaging, medical devices, and predictive algorithms, is consolidated to inform decision-making. Consultation with additional specialists such as psychologists, psychotherapists, nutritionists, and health coaches ensure a holistic approach to patient care.

Development of patient-centric treatment presentation and engagement protocol. Following the assessment provided by the comprehensive health profile, a customized presentation is prepared for the patient. This presentation outlines a tailored recovery strategy and empowers and motivates the patient to actively engage in their health journey. Significant lifestyle adjustments are discussed and implemented through collaborative efforts between the clinic's specialists and the patient. In some cases, the presentation may underscore the necessity of surgical intervention or specialized examinations. After the initial presentation, the doctor-curator and the patient mutually decide whether to proceed with a subscription agreement.

Development of personalized recommendations for nutrition, physical activity, stress management, and other factors are provided based on objective evaluations, anamnesis, and examination results. These tailored guidelines include diet plans, physical activity recommendations, and stress management techniques.

Creating and Upholding a Database System. The Analytical Center actively researches and maintains a database of over 300 esteemed medical institutions and an extensive network of 2000 healthcare professionals for patient referrals and consultations.

Preparation of Analytical Materials. Systematic reviews, clinical and preclinical studies on medical technologies for specific ailments are examined, and digests are compiled and disseminated to healthcare professionals. These materials are also used for organizing scientific seminars.

Compilation of medical updates for physicians. The center collects and summarizes new developments in medical technology, diagnostic methods, and treatments, ensuring doctors have access to the latest insights. Abstracts and analytical reviews on requested topics are also provided.

Synopses of Medical Literature and News Updates. Popular science literature is synthesized into concise abstracts for patients, offering informational support and enhancing patient involvement, compliance, and motivation.

Supporting of communication platform via messenger bot. A messenger bot system facilitates streamlined communication between the clinic and patients. Patients can upload data such as lab results, diet photos, and fitness tracker readings. The system analyzes this information and provides personalized recommendations for further examinations, specialist consultations, and lifestyle adjustments.

### Personnel policy in the proposed longevity clinic framework

The longevity clinic is a unique organization with a unique product, even if it belongs to a network structure. The medical staff and location of the clinic will be unique in each case, and they are key factors in the clinic's effectiveness.

Hiring an international team can benefit a longevity clinic, including access to a diverse talent pool with different perspectives, skills, and cultural backgrounds. This can increase innovation, creativity, and problem-solving capabilities and improve communication and collaboration across global teams.

However, hiring an international team can present challenges such as language barriers, cultural differences, and legal requirements for employing non-citizens. Managing teams across different time zones can also present logistical challenges, and differences in work styles and expectations may need to be addressed. Additionally, extra costs may be associated with relocation, work visas, and language training for international team members.

It's essential to recognize that people from different countries may have different mentalities and work styles, impacting how they approach work and collaboration in a longevity clinic.

Here are some examples:

Communication style: People from some countries may have more direct communication styles, while others may prefer indirect language or avoid confrontation. This can impact how team members give and receive feedback, resolve conflicts, and share ideas.

Work ethic: People from different countries may approach work and productivity differently. Some cultures may value long hours and dedication to work, while others may prioritize work-life balance and taking breaks throughout the day.

Decision-making: In some cultures, decisions are made by a single authority figure, while in others, decisions are made by consensus or after extensive discussion. This can impact how team members make decisions and how they approach problem-solving.

Time management: Cultural differences may exist in how people approach time management and deadlines. Some cultures may prioritize punctuality and meeting deadlines, while others may have a more flexible approach to time.

It's important to recognize and respect these cultural differences and to build a work culture that values diversity and encourages collaboration across different mentalities and work styles. By doing so, you can leverage the strengths of each team member and build a more robust and effective team for your longevity clinic.

### Medical technologies in the longevity clinic

#### Medical technologies and their classification

Longevity clinics employ a mix of established and emerging technologies. These medical technologies encompass a wide range of diagnostic, therapeutic, and preventive tools that address age-related health concerns and optimize longevity outcomes. Medical technologies can be classified based on various criteria, including their validity, reliability, applicability, and based on the principle of function or purpose.

Medical technologies classified based on their primary function and purpose encompass diagnostic technologies, including various imaging modalities like whole-body MRI and PET scans to detect early signs of disease, alongside laboratory tests such as comprehensive blood biomarker panels, whole genome sequencing to analyze DNA and identify disease risks, epigenetic tests to determine biological age, and clinical assessments including physical examinations and cognitive assessments. Some clinically available aging biomarkers are listed in Supplementary Table 4. Treatment technologies include pharmacological treatments like medications, geroprotective supplements use [27-31] (Supplementary Table 5), therapies like physiotherapy to improve fitness (Supplementary Table 6), as well as alternative therapies such as acupuncture and chiropractic care. As an example of non-invasive non-pharmacological therapy, the potential benefits of medical spa treatments in reducing biological age were described as a part of the Kivach Clinic program [32].

Longevity clinics also may offer cutting-edge experimental therapies; this category encompasses innovative approaches like epigenetic reprogramming to reverse cellular aging, senolytics to selectively remove senescent cells, gene therapies, nanomedicine, and treatments such as young blood transfusions and plasma exchange. Some of these technologies hold great promise, for example, the study by Gilmutdinova et al. (2023) [33] demonstrates that therapeutic plasmapheresis effectively reduces aging biomarkers in individuals aged 40-60, highlighting its potential for treating age-related diseases. The procedure showed significant decreases in various biomarkers without adverse reactions, indicating its safety and tolerability. Implementing plasmapheresis in clinical practice could lead to novel treatments for chronic age-related conditions, potentially increasing life expectancy and improving quality of life. Digital health technologies consist of health information systems like electronic health records and telemedicine platforms for video consultations, while health monitoring devices such as wearable fitness trackers and smartwatches enable continuous health monitoring. Additionally, preventive and wellness technologies offer lifestyle interventions like tailored exercise and fitness regimens, nutrition plans based on individual needs, and preventive screenings. Finally, therapeutic technologies encompass regenerative medicine with stem cell therapy, hormone replacement therapy, precision medicine including targeted therapies, and integrative medicine incorporating mind-body therapies and mindfulness-based stress reduction.

Based on validity, diagnostic medical technologies can be evidence-based, like comprehensive blood panels measuring biomarkers of aging and disease risk, or experimental, such as various clocks estimating biological age [34], some of which are presented in Supplementary Table 7. These biological age clocks provide valuable information about an individual's biological age, which may differ from their chronological age. By assessing biological age using these methods, longevity clinics can identify individuals at higher risk of age-related diseases and mortality and develop targeted interventions to slow the aging process and promote healthy longevity. Additionally, these clocks can be used to monitor the effectiveness of interventions over time, helping to optimize personalized treatment plans for everyone.

Regarding reliability, established technologies include electrocardiograms (ECG) for assessing heart health, while developing technologies encompass AI-powered analysis of health data to predict disease risk and guide personalized interventions. Accessibility further divides technologies into those widely available, like blood pressure monitoring, and those with limited availability, such as whole genome sequencing for personalized risk assessments. For treatment technologies, the classifications follow similar lines. Validity includes evidence-based approaches like personalized nutrition plans and experimental methods like young blood plasma transfusions NCT02803554 [35]. Reliability differentiates between established programs, such as targeted physical activity for improving strength and cardiovascular health [36] and developing methods like cryotherapy for inflammation reduction [37, 38]. Applicability ranges from general-purpose stress management therapies to disease-specific treatments like hormone replacement therapy for age-related declines. Lastly, accessibility distinguishes widely available nutritional supplements from emerging technologies like stem cell therapies and regenerative medicine [39].

We would also like to introduce a classification of medical technologies developed by us, based on their market dynamics and adoption trends within the healthcare industry. Based on the performed analysis, we propose to divide medical technologies in the longevity clinic into stable, growing, popular, and vulnerable positions. Each category is accompanied by a detailed characterization and recommendations for their utilization in longevity clinics, as outlined in Supplementary Table 8.

#### Diagnostic and curative longevity programs

The goal of the longevity clinic is to meet the needs of patients and is to achieve active longevity. The technologies used for this may change according to the latest developments in biotechnology. At the same time, it is not certain technologies that are important for the patient, but the achievement of the above-mentioned overall result. That is why we concluded that medical technologies in the longevity clinic should be combined into complexes called programs. Programs can be diagnostic and curative and can combine both components. The programs differ in the tasks to be solved, the depth of diagnosis or correction, as well as the cost.

Longevity clinics offer comprehensive diagnostic programs to assess an individual's health status and biological age. Biological age predictors, validated in large cohorts, show promise for improving health monitoring and life expectancy estimation in clinical practice, though further refinement is necessary for widespread adoption [40]. The general concept behind these functional system check-ups is to provide a comprehensive, data-driven assessment of an individual's health status and aging trajectory. By identifying potential health risks and areas for optimization early on, longevity clinics aim to develop targeted, personalized interventions to improve healthspan and lifespan.

The first step always includes health assessments, conducted by extensive evaluations of medical history, questionaries (Supplementary Table 9), physical examinations, and various clinical tests to detect early signs of age-related functional decline. These assessments may include cognitive, mental, and social interaction evaluations, as well as assessments of sleep quality, nutritional status, and physical performance. Questionnaires provide a comprehensive assessment of psychological well-being, personality traits, quality of life, and loneliness, all of which are important factors to consider in the context of a longevity clinic. By using these tools, clinicians can gain valuable insights into an individual's overall health and well-being and develop personalized interventions to promote healthy aging and longevity.

Our proposed framework encompasses a comprehensive health assessment, covering a wide range of parameters aimed at providing a thorough evaluation of an individual's overall well-being. This assessment delves into various aspects, including functional system check-ups, anthropological markers, metabolic profiles, and anamnesis to gather medical history. Additionally, it evaluates immune system functionality, respiratory health, inflammatory markers, and liver function. Genetic markers and epigenetic influences, alongside assessments of cardiovascular health, microbiota, and digestive system function, are also considered. Psycho-physiological parameters, musculoskeletal health, sensory functions, and psycho-emotional well-being are examined to provide a holistic view of the individual's health. Dental health, daily regime, nasopharynx health, diet, hormonal balance, and physical activity patterns are further evaluated, alongside factors such as cancer risks, stress levels, and blood system health. This proposed frameworkenables tailored health management strategies and facilitates preventive care measures to optimize overall health and well-being.

Examples of diagnostic programs encompassed by our proposed model of a longevity clinic are presented in Supplementary Table 10.

## DISCUSSION

Based on the analysis of various longevity clinics worldwide, it is evident that the field of longevity medicine is rapidly evolving but still faces significant challenges and limitations. The current state can be characterized as a patchwork of cutting-edge diagnostics, experimental therapies, and personalized lifestyle interventions aimed at extending healthspan and lifespan. However, there is a lack of standardization, regulatory oversight, and robust evidence for many of the approaches being utilized. The field of longevity medicine is still evolving, with ongoing debates about the most effective diagnostic tests, interventions, and outcome measures [41, 42]. The landscape of longevity clinics is diverse, ranging from luxury wellness resorts to medically focused centers. Leading clinics (see Supplementary Table 2) combine advanced diagnostics like whole genome sequencing, body imaging, and in-depth blood biomarker analysis with personalized treatment plans. These plans span nutrition, exercise, stress management, supplementation, and sometimes experimental regenerative medicine therapies. However, the field is rife with ethical and scientific concerns, including the marketing of unproven and expensive treatments, risks of unnecessary testing and overdiagnosis, and the use of interventions lacking convincing safety and efficacy data. Regulatory and ethical challenges also need to be addressed [1].

Longevity medicine aims to redefine aging as a treatment condition and shift healthcare from disease treatment to prevention and optimization. It aims to extend healthspan by targeting aging mechanisms and optimizing biological age. ICD-11's classification of aging as a disease supports this approach, fostering advancements in treatments that enhance both healthspan and lifespan [43].

However, this framing remains controversial, with concerns about overpromising and creating unrealistic expectations. Scientifically, while clinics utilize some evidence-based interventions, they also offer an array of experimental diagnostics and therapies backed by minimal clinical research. This underscores the urgent need for further clinical trials and research related to aging. Longevity clinics can play a crucial role in this ecosystem, providing valuable data and serving as sites for clinical trials [44].

Given these limitations, further research is required to establish scientific consensus, develop validated protocols, and consider socioeconomic factors. The Longevity clinic framework, proposed in this study, while offering a detailed concept, may not capture all the alternative approaches and best practices in this rapidly evolving field. As the field matures, it will be crucial to continually reassess and refine the recommendations for building longevity clinics. Future research should focus on evaluating the effectiveness of different clinic models, identifying key success factors, and establishing industry-wide standards.

Artificial intelligence (AI) holds significant potential in the field of longevity, offering advanced diagnostic and predictive capabilities [45]. AI is poised to play an increasingly vital role in the development and operation of longevity clinics in the coming years [46]. By leveraging machine learning algorithms and vast amounts of health data, AI can enable more precise, personalized, and proactive approaches to extending healthspan and lifespan. In longevity clinics, AI can be used to analyze an individual's genetic, epigenetic, and biomarker data to predict their risk of age-related diseases and identify optimal preventive interventions and treatments. AI can also power digital health tools that continuously monitor key health metrics, detect early signs of decline, and provide real-time guidance to patients. Furthermore, AI is accelerating the discovery of new drugs and therapies to target the biological processes of aging. As AI continues to advance, longevity clinics will be able to offer increasingly sophisticated and effective services to help people maintain optimal health and vitality well into old age [47]. The integration of AI into longevity medicine represents a major step towards a future where extended healthy lifespans are accessible to all.

Large Language Models (LLMs) are increasingly being integrated into longevity medicine. LLMs are employed to analyze complex biological data, identify molecular pathways, and develop therapeutic interventions aimed at extending healthy lifespan [48].

As a rule, the concept of the clinic is largely determined by society's needs and investors' interests. We held several negotiations with potential investors, several of which led to successful cooperation and the creation of clinics in the required format. Therefore, we analyzed the current demographic situation and the situation in the global market for medical services and biotechnologies.

Despite fluctuations and declines in overall market conditions, investment in longevity continues to grow steadily. The economic justification and investment potential of longevity clinics are becoming increasingly evident as the global longevity market is projected to reach around $600 billion by 2025. Venture capital investment in longevity clinics more than doubled from 2021 to 2022, reaching $57 billion, with a rapid expansion of new clinics opening across the US, Switzerland, and the UK (www.alliedmarketresearch.com/longevity-and-anti-senescence-therapy-market-A14010).

This growth is driven by several factors. Firstly, the demographic trend of an aging population is a crucial driver of investment in longevity. As the global population ages, there is a growing demand for innovative solutions to age-related diseases and conditions. Secondly, there is increasing recognition of the economic and social benefits of the investment in longevity. Longer and healthier lives can improve productivity, reduce healthcare costs, and increase economic growth. Additionally, a growing awareness of the social and ethical implications in an aging population has led to a greater focus on developing solutions to improve health span and quality of life in later years.

Investors who are interested in longevity clinics come from a variety of backgrounds, including:

Venture capitalists: Venture capitalists are typically interested in investing in high-growth, innovative start-ups. Longevity clinics developing novel therapies, technologies, and business models to improve health span and extend lifespan are often attractive investment opportunities for venture capitalists.

Private equity firms: Private equity firms may be interested in investing in longevity clinics that are more established and have a proven track record of success. These firms may provide funding for expansion or acquisition opportunities.

**Figure 4.**
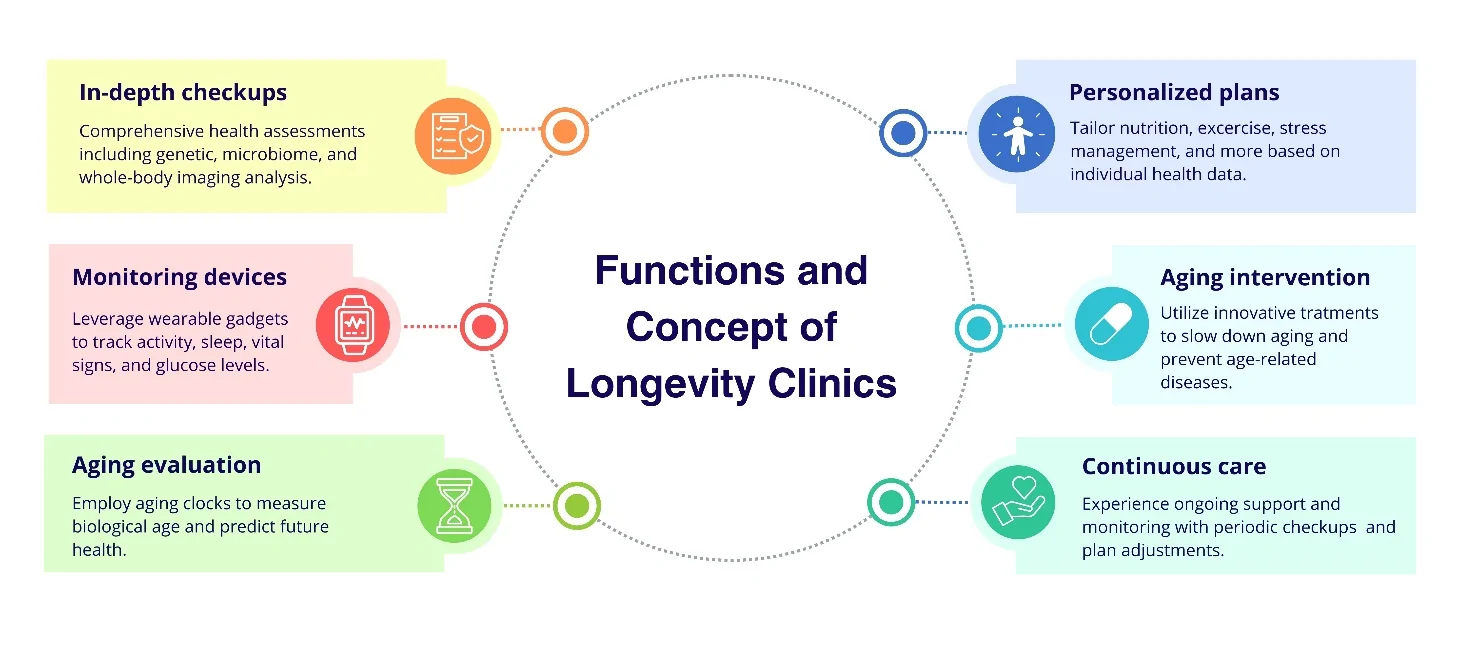
**Functions and Concept of Longevity Clinics**. Longevity clinics provide comprehensive check-ups, utilize monitoring devices, and conduct thorough aging evaluations. They develop personalized care plans based on these evaluations, focusing on preventive and proactive health strategies. Continuous care and regular monitoring ensure that treatment plans are adjusted as needed to optimize patient health and longevity. The clinics also facilitate patient education and engagement, fostering a collaborative approach to health management.

Angel investors: Angel investors are typically high-net-worth individuals who invest in early-stage companies. Angel investors passionate about the potential for longevity clinics to improve health outcomes and extend lifespan may be interested in funding these start-ups.

Corporate investors: Large pharmaceutical companies, health insurers, and other healthcare providers may be interested in investing in longevity clinics to diversify their portfolios and gain exposure to emerging trends in the field.

Philanthropists: Philanthropists passionate about improving public health and promoting social welfare may be interested in funding longevity clinics that are focused on developing therapies and technologies to combat age-related diseases and conditions.

Overall, investors interested in longevity clinics are often motivated by financial and social considerations. They recognize the potential for these clinics to drive innovation, improve health outcomes, and extend lifespan while also providing attractive returns on investment.

In conclusion, longevity clinics offer an innovative solution to the challenges of an aging global population by focusing on preclinical prevention through aging clocks and biomarkers (Fig. 4). Our proposed longevity clinic model integrates a versatile structure with the Analytical Center at its core, driving research and innovation. Clinical units provide specialized services and diagnostics, with a focus on proactive interventions in longevity medicine. Support services ensure smooth operations, reflecting our commitment to personalized care and advancements in longevity science. Our framework ensures flexibility by dividing medical technologies into stable, growing, popular, and vulnerable positions, continuously evaluating and updating them. By centering medical processes around the Analytical Center, longevity clinics can drive innovation, maintain classical diagnostic and treatment methods, and provide high-quality, cost-effective medical services.

Longevity clinics should establish robust cooperation with scientific longevity centers to maximize the efficacy of longevity therapies. By systematically collecting and analyzing biomarker data and assessing the influence of various treatments, clinics can generate valuable datasets for scientific centers. This collaboration enables the comprehensive processing and validation of therapeutic efficacy, creating a feedback loop that enhances intervention precision. Additionally, longevity clinics can serve as pivotal sites for clinical trials, facilitating groundbreaking research and the translation of scientific discoveries into practice. This dual role positions longevity clinics as attractive investment opportunities poised to disrupt traditional healthcare models and significantly enhance human lifespan.

## Supplementary Materials

The Supplementary data can be found online at: www.aginganddisease.org/EN/10.14336/AD.2024.0328.
