# Supplementary material for: A Framework for an Effective Healthy Longevity Clinic
Source: Aging Dis. 2024 Jul 15;16(4):1971–86. doi: 10.14336/AD.2024.0328-1 (PMC12221401; doi:10.14336/AD.2024.0328-1)
Supplement: Supplementary file 1 — The Supplementary data can be found online at: www.aginganddisease.org/EN/10.14336/AD.2024.0328. [file AD-16-4-1971-s.pdf]

SUPPLEMENTARY DATA

A Framework for an Effective Healthy Longevity Clinic

Sergey Mironov, Olga Borysova, Ivan Morgunov, Zhongjun Zhou, Alexey Moskalev

© 2024. Mironov S. et al. Published online at http://www.aginganddisease.org/EN/10.14336/AD.2024.0328-1

SUPPLEMENTARY DATA

Table 1. General Characteristics of Preventive Clinics Established by the Authors

Clinic No	Characteristic	Current state
Clinic 1	Created for a large petrochemical company, focused on preventive treatment for employees, considering their living and working environment. The goal was to maximize staff efficiency and maintain active longevity.	The latest biotechnologies at the time (2017) were implemented over three years.
Clinic 2	Initially established as an aesthetic medicine center, later transformed into an active longevity institute after recognizing the need for an integrated approach and anti-aging technologies.	Continues to operate in this capacity.
Clinic 3	Established during the 2020 coronavirus epidemic by a large company to strengthen client immunity, prevent infectious diseases, and provide restorative treatment after respiratory illnesses.	Despite not primarily focusing on longevity, the clinic utilized longevity medicine technologies for their effectiveness in diagnosis, detoxification, immunity maintenance, and post-illness recovery.
Clinic 4	Opened in 2021 as a longevity clinic, but the management made adjustments to the concept without consulting the authors, implementing only individual project elements in stages.	While achieving good results in preventive medicine, the longevity clinic concept was not fully realized.

Table 2. Longevity Clinics worldwide and their characteristics

Clinic name	Location	Brief description	Diagnostics, Services and Therapies provided	Website
xLongevity	Net of clinics	Network of luxury physical centers providing effective event management and maintaining real-time client access	- Digital client companion - personalized digital interface.<br>- Certified experts - a network of certified doctors and longevity experts.<br>- Supplements and testing - an extensive set of own and third-party supplements and biomarker tests.<br>- Technologies and interventions - 12 integrated longevity technologies. (blood ozonation and oxygenation, hyperbaric oxygen therapy, cryotherapy).	https://xlongevity.com/
Longevity Center	Two clinics (Switzerland, Poland)	Assesses biological age, aging processes, and psycho-emotional state to create personalized longevity plans	- Diagnostics: Measures Metabolic Age, Blood Age, Glycan Age, Neuro Age, Pulmo Age, Cardio Age, various cognitive parameters.<br>- Therapies: Combines state-of-the-art medicine and technology for person-centered care to optimize total health and longevity	https://longevity-center.eu/
Chi Longevity	Singapore	Clinic with comprehensive assessment of biological age and remote coaching	- Initial research (physical examination, medical history, psychological assessment, body composition analysis, genetic testing, cognitive tests, data from wearable devices, biochemical and instrumental checkup, aging clocks).<br>- Psychological assessment methods (Big Five Personality Test, Need for Recovery Scale, Quality of Life Scale, UCLA Loneliness Scale).<br>- Addressing specific health issues, rationalization of supplements, medications and lifestyle, mental health recommendations.	https://www.chilongevity.com/

SUPPLEMENTARY DATA

Human Longevity, Inc	USA, China	Uses AI and advanced diagnostics like whole genome sequencing, imaging, and blood biomarkers to provide precision medicine and personalized care to help clients live longer, healthier lives. Transformation into an ecosystem with its own patient app, collaboration with a stem cell bank, and annual membership.	- Diagnostics: 150GB of data collected per client including 50+ blood biomarkers, whole genome sequencing, whole body MRI.<br>- Therapies: Personalized treatment plans based on in-depth data analysis to prevent diseases of aging.	https://humanlongevity.com/
Fountain Life	USA, UAE	- Focuses on proactive, preventative healthcare using advanced diagnostics and therapeutics<br>- Aims to extend healthspan and help people feel vibrant and healthy to 100+ years old<br>- Uses AI and cutting-edge technologies to detect diseases at their earliest stages<br>- Offers a membership model providing access to longevity physicians, health coaches, diagnostics	- AI-assisted precision medicine and data mining<br>- Exams based on landmark systems biology research<br>- Extracting genetic, chemical, and anatomical biomarkers to build the most comprehensive quantitative snapshot of an individual's health at a point in time<br><br>Diagnostics and treatments:<br>-Full-body MRI with AI to scan for abnormalities<br>-AI coronary artery scan to detect heart disease risks<br>-DEXA bone density scan<br>-Genetic sequencing and epigenetic testing<br>-Gut microbiome analysis<br>-Multi-cancer early detection blood test (50+ cancer types)<br>-Personalized blood panels<br>-Integrated care team of longevity physician, health coaches, nurse practitioners<br>- Researching therapeutics targeting the hallmarks of aging	https://fountainlife.com/
Maximon Longevity Clinic (Ayun)	Switzerland	Digital platform Biolytica, personalized supplements Avea, concierge service for the elderly and offline clinic in Switzerland (plans for a network of clinics worldwide)	- Digital platform for doctor and client with a personalized health program<br>- Supplements unit<br>- Offline clinic in Switzerland (full range of diagnostics, IV therapy, plasmapheresis).	https://www.maximon.com/team
Chenot	Switzerland (flagship location), Azerbaijan, Italy, Montenegro, Morocco	Wellness retreat offering detox and rejuvenation programs based on the Chenot Method	- Development of a methodology for detox and body reboot (nutrition, diagnostics, therapy, lifestyle)<br>- Own laboratory with a focus on genetics<br>- Products for home use	https://www.chenot.com/
Lanserhof	Germany	Scientific laboratory, methodology for working with athletes with injuries, scientific council, R&D, subscription model for patients, methodology,	- Comprehensive health restoration programs on request at the clinic with procedures (hypoxic therapy, cryotherapy).<br>- Gastrointestinal, metabolic, and immune system assessments.<br>- Imaging like ultrasound and echocardiography.	https://lanserhof.com/

© 2024. Mironov S. et al. Published online at http://www.aginganddisease.org/EN/10.14336/AD.2024.0328-1

		partnerships, contract manufacturing	- Specialty tests for genetics, hormones, allergies, sleep, skin, etc.<br>- Therapies and Treatments:<br>- Infusions, physiotherapy, cryotherapy, pain management.<br>- Complementary medicine like acupuncture, osteopathy, craniosacral therapy.<br>- Regenerative and aesthetic treatments<br>- Mental coaching, sleep therapy, stress reduction.	
Sheba Longevity Center (SLC)	Israel	First healthy longevity center as a unit of an academic hospital.	- Diagnostics (lifestyle, biological aging clocks, sensorics, psychology, motor functions, men's and women's health, cognitive functions, body composition, system-oriented approach).<br>- Personalized intervention protocol (S-PIP).<br>- Accessibility, practice in a public facility, affordability, scientific evidence base, AI, data management.<br>- Interdisciplinary scientific research (data science and AI, clinical studies, AI-based drug development, longevity biomarkers, interventional studies, basic science).<br>- Educational programs on longevity.	https://longevity.sheba.co.il/
Healthy Longevity Clinic	Czech Republic and USA	Emphasizes evidence-based interventions to enhance healthy aging. Offers access to cutting-edge therapies and diagnostics.	Diagnostics: Advanced diagnostic tools.<br>Therapies: Brain health, heart health, cellular regeneration, regenerative aesthetics, immune health programs.	https://www.healthylongevity.clinic/
HOOKE London	UK	Focuses on precision medicine and personalized health strategies to promote longevity. Offers some of the world's most advanced health screenings.	Diagnostics: Around 20 assessments including MRI, bone density tests, genomic sequencing, cognitive assessments<br>Therapies: Bespoke nutrition plans, proprietary supplements, lifestyle recommendations.	https://www.hooke.london/
VIMED Medical Longevity Clinic	Poland	Focuses on slowing the aging process to increase healthy lifespan using a unique “Phenotype Therapy approach”.	Diagnostics: Morphology, biochemistry, inflammation, metabolism, nutrigenomics, ultrasound, cell analysis<br>Therapies: Hyperbaric oxygen, laser therapy, nutrition therapy, IV therapy.	https://vimedmlc.com/
Longevity Medical Clinic	USA	Offers longevity programs to preserve health and memory. In operation for over 20 years.	Diagnostics: Body composition analysis, blood tests to evaluate risks of brain shrinkage, Alzheimer's, cancers<br>Therapies: Personalized longevity treatment plans.	https://longevitymedicalclinic.com/
Clinique La Prairie	Switzerland	Renowned clinic offering holistic programs for longevity, well-being and beauty for over 90 years. Uses a four-pillar approach of medicine, nutrition, well-being and movement.	Longevity consultations, sleep quality assessments, DNA tests<br>Therapies: Personalized nutrition, personal training sessions, spa treatments, revitalization programs.	https://cliniquelaprairie.com/

SUPPLEMENTARY DATA

Table 3. Wearable gadgets for self-testing health parameters

Device	Brand	Technical Characteristics	Key Parameters Measured
Apple Watch Series 9	Apple	Sensors:<br>Electrical heart sensor for taking ECGs and measuring heart rate<br>3rd generation optical heart sensor for continuous heart rate tracking<br>Blood oxygen sensor for measuring SpO2 levels<br>Temperature sensing for tracking skin temperature during sleep<br>High-g accelerometer (up to 256 g-forces) and gyroscope for fall detection and crash detection<br>Ambient light sensor for measuring time spent in daylight<br>Chip:<br>S9 SiP with 64-bit dual-core processor and 4-core Neural Engine for powering health monitoring features and on-device processing<br>Connectivity:<br>LTE and UMTS in GPS + Cellular models for connecting to cellular networks and making emergency calls<br>Wi-Fi and Bluetooth 5.3 for connecting to iPhone and wireless accessories<br>Other Specs:<br>Always-On Retina LTPO OLED display for easy visibility of health stats<br>18-hour battery life for all-day tracking<br>50 meter water resistance for swimming and water-based activities<br>32GB storage capacity for saving health data	Heart rate (optical heart sensor, electrical heart sensor for ECG)<br>Irregular heart rhythm notifications<br>ECG (electrocardiogram)<br>Blood oxygen level (SpO2)<br>Sleep tracking (time asleep, sleep stages, respiratory rate)<br>Wrist temperature during sleep (Series 8 and 9)<br>Activity tracking (active calories, steps, distance, flights climbed)<br>Workout tracking (running, hiking, cycling, swimming metrics and more)<br>Fall detection (with Emergency SOS)<br>Noise level monitoring<br>Menstrual cycle tracking<br>Cardio fitness (VO2 max)<br>Mindfulness and breathe sessions
Fitbit Charge Series	Fitbit	Optical heart rate monitor (PurePulse technology) for continuous heart rate tracking<br>Accelerometer and gyroscope for tracking movement, steps, and sleep<br>SpO2 sensor for measuring blood oxygen saturation levels<br>Skin temperature sensor for tracking variations in skin temperature<br>Electrodermal activity (EDA) sensor for stress monitoring (Charge 5)<br>Built-in GPS for tracking pace and distance during outdoor workouts<br>Battery life of up to 7 days (Charge 4) and 5 days with GPS usage<br>Water resistance up to 50 meters	Heart rate (resting heart rate, heart rate zones, heart rate variability)<br>Sleep stages (light, deep, REM) and sleep score<br>Blood oxygen saturation (SpO2)<br>Skin temperature variation<br>Stress levels (Charge 5)<br>ECG for heart rhythm assessment (Charge 5)<br>Breathing rate<br>Steps, distance, calories burned, and active zone minutes<br>Menstrual cycle tracking
Garmin Forerunner Series	Garmin	Optical heart rate monitor (Garmin Elevate) for continuous heart rate tracking<br>Pulse oximeter sensor for measuring blood oxygen saturation (SpO2)<br>Accelerometer for tracking movement, steps, and sleep<br>Built-in GPS (some models with multi-band/dual-frequency GPS) for tracking pace, distance, and routes<br>Altimeter for tracking elevation and stairs climbed<br>AMOLED display on some models (Forerunner 265, 965) for better visibility of health stats	Heart rate (resting, active, HRV)<br>Respiration rate<br>Blood oxygen saturation (SpO2)<br>Stress levels (relax reminders, stress score)<br>Sleep (duration, stages, quality/score)<br>Body Battery energy levels<br>Hydration tracking<br>Menstrual cycle tracking<br>Breathing rate<br>Fitness age<br>VO2 max (aerobic performance capacity)

SUPPLEMENTARY DATA

		Battery life ranging from 7 days to 2 weeks or more depending on model and usage<br>Water resistance for swimming and water-based activities	Training load, status, and effect<br>Recovery time and advisor<br>Performance condition<br>Lactate threshold
Oura Ring	Oura	Sensors:<br>PPG (photoplethysmography) sensor: Infrared LEDs for measuring heart rate, respiration, HRV<br>7 temperature sensors including NTC sensors and advanced calibrated sensor for skin temperature<br>3D accelerometer and gyroscope for tracking movement and daily activities<br>Green and red LEDs for daytime and workout heart rate<br>SpO2 sensor for blood oxygen measurement<br>Material: Titanium with scratch-resistant diamond-like carbon (DLC) coating<br>Inner molding: 100% medical grade, seamless and non-allergenic<br>Weight: 4-6 grams (depending on ring size), lighter than a conventional ring<br>Width: 7.9mm, thickness: 2.55mm<br>Water resistance: Up to 100 meters (328 feet)<br>Battery life: 4-7 days on a single charge, 20–80-minute charging time<br>Memory: Stores data for up to 6 weeks without syncing	Sleep stages (light, deep, REM) and sleep score<br>Resting heart rate, heart rate variability (HRV), respiratory rate<br>Blood oxygen saturation (SpO2) and breathing disturbances during sleep<br>Skin temperature deviations and trends<br>Activity and movement intensity, steps, equivalent walking distance<br>Calories burned, activity contributors<br>Readiness score based on sleep, activity, HRV, body temperature, resting HR<br>Menstrual cycle tracking and prediction
WHOOP Strap	WHOOP	PPG (photoplethysmography) sensor with 5 LEDs (three green, one red, and one infrared) and 4 photodiodes for measuring heart rate, blood oxygen, skin temperature<br>3-axis accelerometer and gyroscope for tracking movement and activity<br>Collects over 100MB of data per day on average, processed on device<br>Battery life of 4-5 days on a single charge, on-wrist charging<br>Water resistance up to 10 meters (IP68)<br>Bluetooth Low Energy (BLE) compatible for connecting to smartphones	Heart rate (live, resting, HRV)<br>Blood oxygen saturation (SpO2)<br>Skin temperature<br>Respiratory rate<br>Sleep stages (awake, light, REM, slow-wave/deep) and sleep efficiency score<br>Strain (cardiovascular load and stress on body)<br>Recovery (readiness to perform based on HRV, RHR, respiratory rate, sleep)
Samsung Galaxy Watch Series	Samsung	BioActive Sensor: Incorporates optical heart rate, electrical heart signal, and bioelectrical impedance analysis sensors into a single chip for extensive health readings<br>Accelerometer, gyroscope, barometer for tracking movement, steps, floors climbed, and sleep<br>GPS for tracking pace, distance, and routes during outdoor workouts<br>Super AMOLED always-on display for easy visibility of health stats<br>Exynos W920 dual-core processor and 1.5GB RAM for fast performance<br>Large battery capacity (e.g. 361 mAh on 44mm Galaxy Watch4) for multi-day usage<br>5ATM + IP68 water resistance for swimming and water-based activities	Heart rate (optical sensor for continuous tracking, electrical sensor for ECG)<br>Blood oxygen saturation (SpO2)<br>Blood pressure<br>Stress levels<br>Sleep stages (light, deep, REM), snoring, blood oxygen during sleep<br>Skin temperature during sleep (Galaxy Watch5 and later)<br>Steps, floors climbed, active time<br>Calories burned<br>Over 100 workout modes with automatic tracking for some activities<br>Women's health and menstrual cycle tracking<br>Body composition (skeletal muscle, fat mass, body fat, BMI, body water)
Amazfit Bip Series	Amazfit	BioTracker PPG optical sensor for heart rate, blood oxygen (SpO2), breathing	Heart rate: 24/7 continuous heart rate tracking, heart rate zones during exercise

		3-axis accelerometer and gyroscope for motion and activity tracking<br>Geomagnetic sensor for positioning<br>Connectivity: Bluetooth 5.0 and above<br>GPS: Built-in GPS + GLONASS on some models (Bip S, Bip 3 Pro) for route tracking<br>Battery Life: Ranges from 15 days (typical usage) to 40+ days (basic usage) on a single charge<br>Water Resistance: 5 ATM water resistance on most models	Blood oxygen saturation (SpO2)<br>Sleep: Sleep stages (deep, light, REM), sleep quality score, breathing quality<br>Stress levels<br>Breathing rate and breathing exercises<br>PAI (Personal Activity Intelligence) score based on activity<br>Steps, distance, calories burned<br>Menstrual cycle tracking<br>60+ sports modes with some auto-detection of workouts<br>Some models support additional metrics like VO2 max
Polar Vantage Series	Polar	Polar Precision Prime sensor fusion technology: Optical heart rate sensor for accurate heart rate tracking, even during swimming<br>Integrated GPS and GLONASS for route tracking and navigation<br>Barometer for altitude and ascent/descent tracking<br>Touchscreen color display (Vantage V) or non-touch display (Vantage M)<br>Lightweight design: Vantage M at 45g, Vantage V Titan at 59g with titanium case<br>Battery life: Up to 40 hours training time (Vantage V), 30 hours (Vantage M)<br>Water resistance up to 30 meters for swimming	Heart rate: Continuous heart rate tracking, resting HR, HR zones, HR max, HRV<br>Running power from the wrist (Vantage V)<br>Pace, distance, route tracking with GPS<br>Sleep tracking: Sleep stages (light, deep, REM), sleep quality, Nightly Recharge recovery measurement<br>Daily activity tracking: Steps, calories, activity goal<br>Training Load Pro: Cardio load status, perceived load, training load<br>Recovery Pro: Daily and long-term recovery levels<br>VO2max fitness level measurement<br>Breathing rate and guided breathing exercises<br>Swimming metrics: Distance, pace, strokes, automatic stroke detection
Dexcom G6 Continuous Glucose Monitor (CGM)	Dexcom	Sensor with 10-day wear time, water-resistant, small and discreet<br>One-touch auto-applicator for easy sensor insertion<br>Transmitter that wirelessly sends data to display device, 3-month battery life<br>Compatible display devices: Dexcom receiver or app on Apple/Android smart devices<br>Bluetooth connectivity to pair with smart devices and some insulin pumps<br>Factory-calibrated, no fingerstick calibration required if sensor code is entered<br>Improved accuracy with 9-10% MARD (mean absolute relative difference)<br>Interoperable with other devices like insulin pumps to create automated insulin delivery system	Real-time interstitial glucose values every 5 minutes<br>Glucose trends and rate of change arrows<br>Customizable high and low glucose alerts and alarms<br>"Urgent Low Soon" predictive alert 20 minutes before glucose is expected to drop to 55 mg/dL<br>Retrospective glucose data and patterns viewable in Dexcom CLARITY software
FreeStyle Libre 2 CGM	Abbott	Sensor size: 35mm diameter, 5mm height, 5g weight<br>Sensor wear time: Up to 14 days<br>Sensor memory: 8 hours of glucose data stored on sensor<br>Sensor application: One-touch applicator for easy self-application<br>Sensor filament: Thin (0.4mm), flexible, sterile fiber inserted just under the skin<br>Sensor readings: Automatically measures glucose every minute, stores data every 15 mins<br>Scanning device: Touchscreen reader device or smartphone app (iPhone/Android)	Real-time interstitial glucose values every minute<br>Ambulatory Glucose Profile (AGP) showing glucose trends and patterns over time<br>Current glucose reading, 8-hour glucose history, glucose trend arrow at each scan<br>Time in Range (TIR), Time Above Range (TAR), Time Below Range (TBR)<br>Glucose variability metrics like standard deviation and coefficient of variation<br>Average glucose and estimated A1c

		Scanning frequency: At least once every 8 hours for a full 24-hour glycemic picture<br>Scanning distance: 1-4 cm from sensor, can scan through clothing<br>Calibration: Factory-calibrated, no fingerstick calibration required<br>Accuracy: 9.2% MARD (mean absolute relative difference) vs. YSI reference<br>Alarms: Optional real-time low and high glucose alarms, and signal loss alarm<br>Water resistance: Up to 1 meter for 30 minutes (sensor is fully disposable)	Hypoglycemia and hyperglycemia episode detection<br>Glucose alarm data (when enabled)
Biostrap EVO	Biostrap	PPG sensor with red and infrared LEDs for measuring heart rate, HRV, SpO2, respiratory rate<br>3-axis accelerometer and gyroscope for activity and sleep tracking<br>Connectivity: Bluetooth for syncing with smartphone app<br>Battery Life: 1-2 days on a single charge with normal usage<br>Water Resistance: Can be worn while swimming<br>Design: Lightweight, slim profile with silicone wristband	Heart Rate: Continuous heart rate tracking, resting heart rate, heart rate variability (HRV)<br>Blood Oxygen Saturation (SpO2)<br>Respiratory Rate<br>Sleep: Sleep stages (light, deep, REM), sleep quality score, total sleep time<br>Activity: Steps, calories burned, activity intensity, 100+ activities recognized<br>Stress and Recovery: Based on HRV, resting heart rate, sleep quality<br>Comprehensive Pulse Analysis: 29 different parameters analyzed for each heartbeat<br>Arterial Health: Measures stiffness index, reflection index related to blood vessel function
Garmin Vivosmart Series	Garmin	Display: OLED touchscreen display, size varies by model (e.g. 0.41" x 0.73" on Vivosmart 5)<br>Sensors:<br>Optical heart rate sensor (Garmin Elevate) for continuous HR tracking<br>Pulse oximeter for blood oxygen saturation (SpO2) monitoring<br>Accelerometer and gyroscope for activity and sleep tracking<br>Battery life: Up to 7 days in smartwatch mode (Vivosmart 5)<br>Water resistance: Swim (up to 50 meters)<br>Connectivity: Bluetooth for smartphone notifications and syncing<br>Memory: Stores activity data for up to 7 days between syncs	Heart rate: 24/7 heart rate monitoring, resting heart rate, abnormal HR alerts<br>Blood oxygen saturation (SpO2) - spot checks, during sleep, all-day (varies by model)<br>Respiration rate (24/7)<br>Stress level (all-day stress tracking)<br>Sleep: Sleep stages (light, deep, REM), sleep score, total hours of sleep, movement<br>Body Battery energy level<br>Hydration tracking<br>Menstrual cycle tracking<br>Fitness age<br>Steps, distance, calories burned, intensity minutes<br>Floors climbed (some models)
Xiaomi Mi Band Series	Xiaomi	PPG optical heart rate sensor for continuous heart rate tracking<br>3-axis accelerometer and 3-axis gyroscope for activity and sleep tracking<br>SpO2 sensor for blood oxygen monitoring (Smart Band 6 and later)<br>Battery life: Ranges from 14 days (Smart Band 6, 7) to 16 days (Smart Band 8) on a single charge<br>Water resistance: 5 ATM (up to 50 meters) on most models<br>Connectivity: Bluetooth (4.0 on Mi Band, 5.0 and later on newer models)	Heart rate: Continuous heart rate tracking, resting heart rate, heart rate zones, alerts<br>Blood oxygen saturation (SpO2) - introduced in Mi Band 6<br>Sleep tracking: Sleep stages (deep, light, REM), sleep score, sleep breathing quality<br>Stress monitoring and breathing exercises<br>Female health and menstrual cycle tracking<br>PAI (Personal Activity Intelligence) score based on activity<br>Steps, distance, calories burned

			30+ sports modes with auto-detection of some activities like walking, running, cycling<br>Some models support VO2 max, recovery time, training effect

Key Parameters Explained:
- Heart Rate: The number of heart beats per minute.
- ECG (Electrocardiogram): A measure of the electrical activity of the heart.
- Blood Oxygen (SpO2): The percentage of oxygen in your blood.
- Sleep Quality: Analysis of sleep patterns and stages (light sleep, deep sleep, REM).
- HRV (Heart Rate Variability): The variation in time between heartbeats.
- Steps: Number of steps taken.
- Calories Burned: Estimated calories burned based on activity.
- Activity Tracking: Monitoring various physical activities such as walking, running, cycling.
- Stress Management: Metrics related to stress levels and relaxation.
- Skin Temperature: Measurement of skin temperature changes.
- VO2max: Maximum oxygen uptake during intense exercise.
- Continuous Blood Glucose Monitoring: Real-time monitoring of blood glucose levels.

Table 4. Some clinically available biomarkers, that are measured in Longevity Clinics

**Biomarker**	**Test type**	**Optimal Range**	**Periodicity of Measurement**	**Relevance to Longevity Clinic**
Complete Blood Count (CBC)	Blood Test	Varies by component (e.g., WBC: 4.5-11 x10^9/L, RBC: 4.5-5.9 x10^12/L)	Annualy	CBCs contain robust aging signals [49].<br>Certain CBC components, such as red blood cell distribution width (RDW), have emerged as promising biomarkers for identifying individuals at heightened risk of age-related diseases and mortality [50].
Fasting Glucose	Blood Test	70-99 mg/dL	Annualy	Fasting glucose levels tend to increase with age, and this age-related increase is associated with higher mortality risk.<br>Higher fasting glucose levels, even below the diabetic range, are associated with greater biological age and increased all-cause mortality risk [51-52].
Hemoglobin A1c (HbA1c)	Blood Test	<5.7%	Annualy	HbA1c levels tend to increase with age, even in non-diabetic individuals [53].<br>Elevated HbA1c is a strong risk factor for developing age-related chronic diseases like cardiovascular disease, kidney disease, and dementia [54].<br>Higher HbA1c levels, even within the non-diabetic range, are associated with increased all-cause mortality risk [55].
Lipid Profile	Blood Test	Total Cholesterol: <200 mg/dL, LDL: <100 mg/dL, HDL: >60 mg/dL, Triglycerides: <150 mg/dL	Annualy	Lipid profiles in early adulthood are associated with epigenetic aging in midlife. Elevated triglycerides and low HDL cholesterol in young adults correlate with accelerated epigenetic aging, an indicator of biological age, assessed decades later [56].

				Lipid profiles can predict CVD risk and all-cause mortality risk in older adults [57].
High-Sensitivity C-Reactive Protein (hs-CRP)	Blood Test	<1.0 mg/L	Annualy	Hs-CRP levels tend to increase with age whichis associated with "inflammaging" [58, 59].<br>Higher hs-CRP levels are predictive of cardiovascular disease [60].
Homocysteine	Blood Test	5-15 µmol/L	Annualy	Older adults typically have higher homocysteine concentrations compared to younger people, which is associated with a disease development [61].<br>Each 5 µmol/L increase in homocysteine may raise all-cause mortality risk by 27-34% [62].
Vitamin D (25-hydroxyvitamin D)	Blood Test	30-50 ng/mL	Annualy	Low vitamin D levels, even within the normal range, are associated with increased all-cause mortality risk in older adults [63].<br>Vitamin D deficiency is linked to many age-related chronic diseases including cardiovascular disease, cancer, cognitive impairment, dementia, osteoporosis, and eye disorders [64].
Thyroid-Stimulating Hormone (TSH)	Blood Test	0.4-4.0 mIU/L	Annualy	Subclinical hypothyroidism, defined as elevated TSH with normal free T4, becomes increasingly common with age [65].<br>Centenarians and people with exceptional longevity tend to have higher TSH levels compared to younger controls [66].
Insulin	Blood Test	2-25 µIU/mL	Annualy	Insulin levels tend to increase with age, even in non-diabetic individuals. This age-related increase in insulin is thought to contribute to the development of insulin resistance and various age-related diseases [67].<br>Elevated insulin levels, even within the normal range, are associated with increased all-cause mortality risk in older adults. The optimal insulin range for longevity appears to be on the lower end of normal<br>Insulin resistance is a major risk factor for many age-related diseases including type 2 diabetes, cardiovascular disease, cognitive decline, and certain cancers [68].
Creatinine	Blood Test	0.7-1.3 mg/dL (men), 0.6-1.1 mg/dL (women)	Annualy	Creatinine clearance, a measure of kidney function, decreases with age [69].<br>People who lived to 100 tended to have lower levels of creatinine from their 60s onwards compared to those who did not become centenarians.
Blood Urea Nitrogen (BUN)	Blood Test	7-20 mg/dL	Annualy	BUN levels increase with age in both men and women up until around 70-86 years old [70].<br>BUN is a surrogate marker for neurohormonal activation, which is thought to be central to the pathophysiology of heart failure in the elderly [71].

Alanine Aminotransferase (ALT)	Blood Test	7-56 U/L	Annualy	In a longitudinal study, ALT decreased by 10-20% over 8-15 years in older adults [72].<br>In the elderly, low ALT is associated with frailty, sarcopenia, disability, and reduced survival [73].
Aspartate Aminotransferase (AST)	Blood Test	10-40 U/L	Annualy	AST levels tend to increase slightly with age in both men and women, with the elderly having higher normal ranges than younger adults [74].<br>In a longevity study, people who lived to 100 tended to have lower AST levels from their 60s onwards compared to those who did not become centenarians [52].
Gamma-Glutamyl Transferase (GGT)	Blood Test	9-48 U/L	Annualy	In longevity studies, people who lived to 100 tended to have lower GGT levels from their 60s onwards compared to those who did not become centenarians [52].<br>GGT is a sensitive marker of oxidative stress, and its levels are associated with various age-related diseases such as cardiovascular disease, metabolic syndrome, and fatty liver disease [75].
Albumin	Blood Test	3.5-5.0 g/dL	Annualy	Albumin levels decrease with age in both men and women, with the decline starting around age 70.<br>Lower albumin levels in older age are associated with increased all-cause mortality, frailty, sarcopenia, and disability [76].<br>The higher albumin in the older centenarian group may reflect better overall health status and reduced oxidative stress [52].<br>Albumin has multiple physiological roles including transport, antioxidant activity, and maintaining oncotic pressure. Reduced albumin with age may impair these functions and contribute to aging-related pathologies [77].
Ferritin	Blood Test	20-500 ng/mL (men), 20-200 ng/mL (women)	Annualy	Ferritin levels tend to increase with age in both men and women, showing an age-related rise up until around 70-80 years old. This increase is thought to be a consequence of the activation of the reticuloendothelial system and increased iron storage with aging [78].<br>The lower ferritin in this older age group may reflect better overall health status and reduced inflammation.<br>Elevated ferritin levels in the elderly, even within the normal range, are associated with increased all-cause, cardiovascular, and liver disease mortality [79].
Interleukin-6 (IL-6)	Blood Test	<2 pg/mL	Annualy	Age-related increase in IL-6 is thought to contribute to the chronic low-grade inflammation observed in older adults, a phenomenon known as "inflammaging".<br>Elevated IL-6 levels in the elderly are associated with increased risk of age-related diseases, frailty, disability, and mortality [80].

				Potential mechanisms linking IL-6 to aging include its role in chronic inflammation, oxidative stress, and cellular senescence [81].
Tumor Necrosis Factor-alpha (TNF-alpha)	Blood Test	<8.1 pg/mL	Annualy	TNF-α levels increase with age in both humans and animal models. This age-related increase in TNF-α is thought to contribute to chronic low-grade inflammation or "inflammaging" observed in older adults. Elevated TNF-α in aging is associated with increased risk of age-related diseases, frailty, sarcopenia, and mortality [82, 83].
Telomere Length	Blood Test	Age-adjusted	Every few years	Telomere length has been proposed as a biomarker of biological aging, as shorter telomeres are associated with increased age and age-related diseases [84].<br>Cross-sectional studies show that leukocyte telomere length (LTL) decreases with age, with an average attrition rate of 20-40 base pairs per year [85].
Bone Mineral Density (BMD)	Functional Test	T-score > -1.0	Every 2 years	BMD decreases with age in both men and women, with the decline accelerating after age 50. Women experience a more rapid decline in BMD compared to men, especially during the menopausal transition and early postmenopausal years. The age-related decrease in BMD is associated with increased risk of osteoporosis, fractures, and mortality [86].
Cognitive Function Tests	Functional Test	Age-adjusted	Annualy	Cognitive function tests can detect age-related cognitive decline and serve as biomarkers of brain aging [87].
Grip Strength	Functional Test	Age-adjusted	Annualy	Grip strength is a reliable measure of overall muscle strength and biological vitality that declines with age [88].<br>Higher grip strength in older age may be a marker of successful aging and longevity [89].
Gait Speed	Functional Test	>1 m/s	Annualy	Slower gait speed is associated with increased risk of adverse outcomes such as frailty, disability, cognitive decline, falls, hospitalizations, and mortality [90].
VO2max	Cardiopulmonary Exercise Test (CPET) or Field Test (e.g., treadmill or cycle ergometer)	Age-adjusted (e.g., >35 mL/kg/min for men, >30 mL/kg/min for women, varies by age)	Annually or Biannually	VO2max declines with age starting around 30-40 years old at a rate of about 10% per decade [91].<br>Age-related decline in VO2max is associated with increased risk of all-cause and disease-specific mortality.<br>VO2max is considered one of the best predictors or biomarkers of longevity [92]
Bioimpedance Analysis (BIA) or Dual-Energy X-ray	Body Composition Analysis	Varies by component (e.g., Body Fat Percentage: Men:	Annually or Biannually	Aging is associated with characteristic changes in body composition that can be measured by DEXA:

Absorptiometry (DEXA)		10-20%, Women: 18-28%)		Increase in total and visceral fat mass, especially after age 70 [93];<br>Decrease in lean mass and skeletal muscle mass [94].

Table 5. Geroprotective Supplements (Data sourced from Geroprotectors (http://geroprotectors.org/) and DrugAge (https://genomics.senescence.info/drugs/) Databases)

Name	Geroprotective Mechanisms	Effective Daily Dosage	Cycle
5-HTP	Serotonin precursor, mood regulation	100-300 mg	Continuous
GABA	Neurotransmitter, reduces stress and anxiety	500-1000 mg	Continuous
L-ergothioneine	Antioxidant, protects against oxidative stress	5-10 mg	Continuous
NMN	NAD+ precursor, enhance cellular energy metabolism	250-500 mg	Continuous
NR	NAD+ precursor, enhance cellular energy metabolism	300-500 mg	Continuous
N-acetylglucosamine	Anti-inflammatory, supports joint health	500 mg	Continuous
N-acetylcysteine	Antioxidant, replenishes glutathione levels	600-1800 mg	Continuous
PQQ	Mitochondrial biogenesis, antioxidant	10-20 mg	Continuous
Q10	Antioxidant, supports mitochondrial function	100-200 mg	Continuous
TUDCA	Liver protection, supports cellular homeostasis	250-500 mg	Cyclic (2-3 months on/off)
Alanin	Amino acid, supports muscle metabolism	2-5 g	Continuous
Alpha-ketoglutarate	Krebs cycle intermediate, supports energy metabolism	1-3 g	Continuous
Alpha lipoic acid	Antioxidant, supports mitochondrial function	300-600 mg	Continuous
Arginine	Nitric oxide precursor, improves blood flow	3-6 g	Continuous
Astaxanthin	Potent antioxidant, anti-inflammatory	4-12 mg	Continuous
Berberine	AMPK activator, supports metabolic health	500 mg 2-3x daily	Cyclic (2-3 months on/off)
Beta-hydroxybutyrate	Ketone body, supports energy metabolism	10-12 g	Continuous
Vitamin D	Supports bone health, immune function	2000-4000 IU	Continuous
Hydroxytyrosol	Antioxidant, anti-inflammatory	5-20 mg	Continuous
Ginestein	Phytoestrogen, supports cardiovascular health	50-100 mg	Continuous
Glycine	Amino acid, supports collagen synthesis	3-5 g	Continuous
Glucosamine	Supports joint health	1500 mg	Continuous
DHEA	Hormone precursor, supports hormonal balance	25-50 mg	Cyclic (under supervision)
Isoquercetin	Antioxidant, anti-inflammatory	250-500 mg	Continuous
Carnosine	Antioxidant, anti-glycation	500-1000 mg	Continuous
Catechins	Antioxidant, supports cardiovascular health	200-500 mg EGCG	Continuous
Quercetin	Antioxidant, anti-inflammatory	500-1000 mg	Cyclic (2-3 months on/off)
Caffeic acid	Antioxidant, anti-inflammatory	200-400 mg	Continuous
Curcumin	Anti-inflammatory, antioxidant	500-2000 mg	Continuous
Lecithin	Supports brain health, emulsifier	1-2 g	Continuous

Lysine	Amino acid, supports collagen synthesis	1-3 g	Continuous
Lycopene	Antioxidant, supports cardiovascular health	10-20 mg	Continuous
Lithium	Neuroprotective at low doses	1-5 mg (low-dose)	Continuous (low-dose)
Lutein + zeaxanthin	Supports eye health	Lutein: 10 mg, Zeaxanthin: 2 mg	Continuous
Magnesium citrate	Supports muscle and nerve function	200-400 mg	Continuous
Menaquinone (K2)	Supports bone and cardiovascular health	100-200 mcg	Continuous
Myricetin	Antioxidant, anti-inflammatory	100-400 mg	Cyclic (2-3 months on/off)
Nicotinamide	NAD+ precursor, supports cellular energy metabolism	500-1000 mg	Continuous
Pinitol	Insulin mimetic, supports metabolic health	600 mg	Continuous
Polydatin	Antioxidant, supports cardiovascular health	20-40 mg	Continuous
Proanthocyanidins	Antioxidant, supports cardiovascular health	50-200 mg	Continuous
Proline	Amino acid, supports collagen synthesis	500 mg	Continuous
Rutin	Antioxidant, supports vascular health	500 mg	Continuous
Selenium	Antioxidant, supports immune function	100-200 mcg	Continuous
Serin	Amino acid, supports cognitive function	500 mg	Continuous
Silymarin	Liver protection, antioxidant	240 mg	Continuous
Spermidine	Autophagy induction, cellular regeneration	1–2mg	Continuous
Taurine	Antioxidant, supports cardiovascular health	500–2000mg	Continuous
Theanine	Neuroprotective, stress reduction	200–400mg	Continuous
Trehalose	Autophagy induction	1–3g	Continuous
Uridine	Supports brain function	250–500mg	Continuous
Urolithin A	Mitochondrial biogenesis	250–500mg	Continuous
Ursolic acid	Antiinflammatory	150–300mg	Continuous
Fisetin	Senolytic activity	100–300mg	Cyclic (intermittent)
Zinc citrate	Antioxidant, supports immune function	15–30mg	Continuous
Citrulline	Nitric oxide precursor	3–6g	Continuous

Table 6. Physiotherapy and Regenerative Medicine Approaches to Improve Fitness

Therapy	Mechanisms of Action
Hypoxytherapy (Intermittent Hypoxic Training)	Simulates high-altitude conditions to improve cardiovascular and muscular endurance
Hyperbaric Oxygen Therapy (HBOT)	Involves breathing pure oxygen in a pressurized room or chamber to promote healing and recovery
Cryotherapy	Exposes the body to extremely cold temperatures for a short period to reduce inflammation and speed up recovery
Photobiomodulation (Low-Level Laser Therapy)	Uses red or near-infrared light to stimulate cellular activity, reduce pain, and promote tissue repair
Electrical Muscle Stimulation (EMS)	Uses electrical impulses to contract muscles, enhancing strength, endurance, and recovery
Ultrasound Therapy	Uses high-frequency sound waves to promote tissue healing and reduce pain
Shockwave Therapy	Utilizes acoustic waves to stimulate healing in tendons, muscles, and other soft tissues

Magnetotherapy	Applies magnetic fields to the body to improve circulation and reduce pain
Hydrotherapy (Aquatic Therapy)	Uses water exercises to improve strength, flexibility, and cardiovascular health while reducing joint stress
Manual Therapy	Includes techniques like massage, joint mobilization, and manipulation to improve mobility and reduce pain
Therapeutic Exercise	Tailored exercise programs designed to improve strength, flexibility, endurance, and overall fitness
Kinesio Taping	Uses elastic tape applied to the skin to support muscles and joints without restricting movement
Dry Needling	Involves inserting fine needles into trigger points in muscles to relieve pain and improve function
Therapeutic Ultrasound	Uses sound waves to treat deep tissue injuries by promoting blood flow and reducing inflammation
Laser Therapy	Utilizes specific wavelengths of light to penetrate tissues and promote healing at the cellular level
Pulsed Electromagnetic Field Therapy (PEMF)	Uses electromagnetic fields to stimulate cellular repair and improve circulation
Biofeedback	Teaches control over physiological functions by providing real-time feedback on muscle activity, heart rate, etc
Heat Therapy (Thermotherapy)	Uses heat packs or infrared lamps to relax muscles and increase blood flow to affected areas
Cold Laser Therapy	Employs low-intensity lasers to stimulate healing processes in tissues
Compression Therapy	Uses compressive garments or devices to improve circulation and reduce swelling
Graston Technique	A form of manual therapy that uses specialized instruments to detect and treat soft tissue injuries
Cupping Therapy	Involves placing cups on the skin to create suction, believed to improve blood flow and reduce muscle tension
Proprioceptive Neuromuscular Facilitation (PNF)	Advanced stretching techniques aimed at improving flexibility and strength through proprioceptive input
Balance and Coordination Training	Exercises designed to improve proprioception, balance, and coordination, often using tools like balance boards or stability balls
Functional Movement Screening (FMS)	A systematic approach to evaluate movement patterns and identify limitations or asymmetries that could lead to injury
Stem Cell Therapy	Uses stem cells to promote regeneration of damaged tissues
Platelet-Rich Plasma (PRP) Therapy	Involves injecting concentrated platelets from the patient's own blood into injured a
Regenerative Medicine Techniques	Includes various methods aimed at regenerating damaged tissues, such as tissue engineering and growth factor therapies
Neurofeedback	A type of biofeedback that focuses on brainwave activity to improve mental focus and relaxation
Virtual Reality Rehabilitation	Uses virtual reality environments for physical therapy exercises, enhancing engagement and effectiveness

Table 7. Biological Age Clocks

**Biological Age Clock**	**Measures**	**Relevance to Longevity Clinic**
PhenoAge	Biological age based on nine biomarkers, including inflammation and glucose metabolism	Predicts all-cause mortality and age-related diseases
DunedinPACE (Pace of Aging)	Rate of aging based on changes in 19 biomarkers across multiple organ systems	Identifies individuals aging faster than their chronological age

© 2024. Mironov S. et al. Published online at http://www.aginganddisease.org/EN/10.14336/AD.2024.0328-1

Deep Longevity Blood Age	Biological age based on deep learning analysis of blood biochemistry	Provides a comprehensive assessment of biological age using routine blood tests
Klemera–Doubal method	Biological age based on a set of biomarkers, such as blood pressure and cholesterol	Offers a simple and accessible method for estimating biological age
GrimAge	Biological age based on DNA methylation patterns and smoking history	Predicts time to death, time to cancer, and time to coronary heart disease
Telomere length	Biological age based on the length of telomeres, which shorten with age	Shorter telomeres are associated with increased risk of age-related diseases and mortality
Frailty Index	Accumulation of health deficits across multiple domains, including physical, cognitive, and social functioning	Predicts mortality, disability, and hospitalization in older adults
ipAGE (inflammation-protected age)	Biological age based on the balance between pro-inflammatory and anti-inflammatory cytokines	Predicts mortality risk and identifies individuals with accelerated aging due to chronic inflammation
Cognitive Clock	Biological age based on cognitive function, including memory, attention, and processing speed	Identifies individuals with accelerated cognitive aging and increased risk of dementia

Table 8. Classification of medical technologies developed by us, based on their market dynamics and adoption trends within the healthcare industry

Category	Characteristics	Examples	Advantages	Disadvantages	Recommendations
Stable positions	- Stable demand, predictable revenue.<br>- Consistent usage by doctors and patients.<br>- Unaffected by external factors.	Consultations with general practitioners.<br>Functional diagnostics of cardiovascular and respiratory systems.<br>Basic laboratory diagnostics, etc.	High predictability, stability, possibility of expansion.	- High prevalence and moderate profitability.<br>- Average market price with a moderate yield (usually 5-10%).<br>- Impractical to advertise due to widespread recognition and moderate profit margins.	Necessity: These services cannot be excluded from the clinic's offerings.<br>Revenue Share:<br>- Innovative Clinics: Up to 30% of revenue from stable services;<br>- Stable Business Clinics: Up to 95% of revenue from stable services<br>Strategy: The share of stable services in the revenue structure should align with the clinic's business model, whether focused on innovation and development or stable, predictable growth.

SUPPLEMENTARY DATA

Growing positions	- Growth driven by medical advancements or epidemics.<br>- Requires limited popularity initially.<br>- Advertising leveraged for promotion.<br>- Allows for high pricing (up to 100% or more).<br>- Additional costs for training and evaluating effectiveness.		- High profitability.<br>- Additional motivation of the most ambitious doctors	- Additional costs for marketing, training, and research.<br>- Limited period of growth: Effective technology spreads to most clinics, while inefficient ones cease distribution	- Ambitious clinics lead in adopting growing technologies.<br>- Startups heavily reliant on such technologies.<br>- Suggested revenue share limit of 50% for balanced development.<br>Emphasis on investing in doctor training, internal research, and marketing.<br>- Continual search for new technologies in case of inefficiency in existing ones
Popular positions	- Services characterized by growth, primarily driven by marketing and advertising.<br>- Public opinion amplifies real achievements in medical science.<br>- Often based on well-known technologies without breakthroughs, like nutraceuticals and cosmetology.<br>- Most doctors in the specialty understand and may own the service.<br>- Patients seek the best rather than unique service; competition among marketers.<br>- Marginality typically capped around 50% due to competition and marketer effectiveness.		High marginality with minimal investment	- High competition typical.<br>- Patients and doctors often have high expectations.<br>- Risk of technology becoming unpopular over time, especially if lacking serious scientific basis.<br>- Attracts doctors seeking high personal income rather than scientific motivation.	- Consider allocating these services to a separate unit or clinic dedicated solely to this area.<br>- Prioritize active investment in marketing to maintain popularity.<br>- Develop contingency plans for winding down the business if service popularity declines.
Vulnerable positions	- Vulnerable positions in clinics yield the best results with proper management.<br>- Typically, these are well-known but unpopular services due to various factors like complexity or availability of alternatives.	- Sections of homeopathy.<br>- Hydrocolonotherapy<br>- Hirudotherapy.	- Low competition offers the opportunity to occupy a unique niche in the market.<br>- High patient compliance and doctor	- Low income.<br>- Reputational risks.<br>- Lack of scientific development prospects.<br>Rarity of doctors offering such services, with young and ambitious doctors rarely involved.	- Engage in vulnerable technologies if interested patients and capable doctors are available, and the niche is open in the local market.<br>- Limit the share of these services in a multidisciplinary clinic to 5%.<br>- Consider separating this area into a distinct business to

	- Large-scale scientific research on these technologies is often lacking.<br>- Enthusiastic doctors and patients sympathetic to these technologies are typically involved.<br>- Low competition due to vulnerability, but marginality usually capped around 10% as patients are unwilling to overpay.		loyalty to the clinic.<br>- Income stability and predictability typically low but consistent.		mitigate potential reputational risks.

Table 9. Questionaries, used by Longevity Clinics to estimate the Quality of life and psychological state

Questionnaire Name	Measures	Relevance to Longevity Clinic
SF-36	General health status and quality of life	Assesses physical, mental, and social well-being
Montreal Cognitive Assessment (MoCA)	Cognitive function, including memory and executive function	Screens for mild cognitive impairment and early Alzheimer's disease
Well-Being Questionnaire (WAM)	Current well-being, including self-feeling, activity, and mood	Monitors psychological state and overall well-being
Prime Diet Quality Score (PDQS)	Quality of dietary intake	Evaluates nutrition as a key factor in healthy aging
Healthy Lifestyle Profile	Stress, anxiety, and depression (HADS scale)	Identifies psychological factors that may impact longevity
The Big Five Personality Test	Openness, Conscientiousness, Extraversion, Agreeableness, Neuroticism	Determines readiness to engage in health-improving activities
The Need for Recovery Scale	Work-related fatigue and need for recovery	Assesses the impact of work on well-being and recovery needs
The Health-Related Quality of Life Scale (5Q-5D-5L)	Functioning in five aspects of quality of life and self-rated health	Evaluates overall health state, outcomes, and quality of life
The UCLA Loneliness Scale Version 3	Experiences of loneliness, quality of relationships, and social connections	Assesses the impact of social factors on mental health and longevity

SUPPLEMENTARY DATA

Table 10. Comprehensive Diagnostic and Curative Program Examples in our Longevity Clinic Model

Name of the Program	Characteristics	Tests included	Therapy options
**Comprehensive Diagnostic Programs**			
Basic program	Standard check-up for determining the indicators of the functioning of the most important organs and systems, as well as the early detection of the most common and dangerous diseases. Includes basic instrumental and laboratory studies.	Clinical and biochemical studies of blood<br>Examination of thyroid gland function<br>Analysis of main vitamins (C, A, E, D3, B9, B12)<br>Assessment of trace elements (zinc, selenium, copper, iron, calcium)<br>Measurement of blood coagulation rates<br>Estimation of total antioxidant activity<br>Coprogram to assess gastrointestinal tract function	-
Balanced program	Check-up allows to identify not only the most common, but also more rare disorders, as well as to give accurate recommendations on lifestyle, nutrition and medical procedures for active longevity.<br>Vital for diagnosing diseases related to environmental factors, poor nutrition, and hereditary conditions	Expanded content of vitamins and trace elements<br>Comprehensive cancer marker panel<br>Diagnosis of infectious agents (*H. pylori, Mycoplasmas, Chlamydia, Hepatitis viruses, Candida*)<br>Tests for optimal nutritional parameters	-
Full program	Increased scope of diagnostics with complex and high-tech laboratory studies	Microelements: aluminum, cadmium, cobalt, mercury, lead, manganese, arsenic, nickel<br>Interleukin levels<br>Identification of rheumatic diseases<br>Determination of catecholamines in blood and urine: adrenaline, norepinephrine, dopamine	-
**Curative Targeted Programs**			
MindSpan: Cognitive Health Longevity Program	The program targets adults aged 40 and older, focusing on optimizing cognitive health, preventing age-related mental decline, and	Comprehensive Cognitive Assessment:<br>- Memory tests (short-term and long-term)	Cognitive Training:<br>- Personalized brain exercises and memory enhancement techniques

	addressing early signs of cognitive impairment or risk of neurodegenerative diseases. It also supports those experiencing stress, anxiety, or depression related to aging and aims to enhance overall mental well-being as part of a holistic longevity approach.	- Attention and concentration evaluations<br><br>- Executive function assessments<br><br>- Language skills tests<br><br>Neuroimaging:<br><br>- MRI brain scan to assess brain structure and detect any abnormalities<br><br>- fMRI to evaluate brain activity patterns<br><br>Biomarker Analysis:<br><br>- Blood tests for inflammation markers, hormones, and nutrient levels<br><br>- DNA methylation tests to analyze aging-associated genes<br><br>Metabolic Health Monitoring:<br><br>- Regular blood glucose and insulin level testing<br><br>- Continuous glucose monitoring for personalized dietary insights<br><br>Psychological Evaluation:<br><br>- Standardized questionnaires for depression, anxiety, and stress<br><br>- Quality of life assessments<br><br>Sleep Study:<br><br>- Polysomnography to assess sleep quality and patterns	- Computer-based cognitive training programs<br><br>Nutritional Therapy:<br><br>- Personalized diet plans focusing on brain-healthy foods<br><br>- Anti-Inflammatory Diet<br><br>- Targeted supplementation for cognitive support<br><br>Physical Exercise Program:<br><br>- Tailored exercise routines to promote brain health<br><br>- Mind-body exercises like tai chi or yoga<br><br>Insulin Sensitivity Optimization:<br><br>- Incorporate regular exercise, especially fasted cardio and strength training<br><br>- Consider metformin or other insulin-sensitizing medications under medical supervision<br><br>Gut Microbiome Optimization:<br><br>- Probiotic and prebiotic supplementation<br><br>- Dietary changes to support a healthy gut-brain axis<br><br>Hormetic Stress Exposure:<br><br>- Controlled exposure to mild stressors like heat (sauna) or cold therapy to potentially activate longevity pathways

			Social Engagement Activities:<br>- Group workshops and classes to promote social interaction<br>- Community involvement programs<br>Sleep Optimization<br>Nutritional support and supplements (curcumin, omega-3s, etc.)
Hormonal Harmony: Female Program	The program targets women in their 40s and older, focusing on peri-menopausal women experiencing symptoms like irregular periods, hot flashes, mood swings, and sleep disturbances, as well as postmenopausal women managing long-term health concerns.	Comprehensive hormone panel (blood test)<br>Dried Urine Test for Comprehensive Hormones) for hormone metabolites<br>Genetic testing for hormone metabolism<br>Bone density scan (DEXA)<br>Thyroid function tests<br>Body composition analysis<br>Lipid profile for cardiovascular risk assessment<br>Fasting blood glucose and HbA1c for diabetes screening<br>Mammogram and breast exam	Personalized hormone therapy (if appropriate):<br>- Estrogen therapy<br>- Combination therapy (estrogen and progesterone)<br>- Bioidentical hormone options.<br>Lifestyle interventions:<br>- Nutrition counseling and personalized diet plans<br>- Exercise programs tailored for women<br>- Stress management techniques (e.g., meditation, yoga)<br>Supplements and nutraceuticals:<br>- Personalized vitamin and mineral supplementation<br>- Herbal remedies (e.g., black cohosh, evening primrose oil)<br>Vaginal microbiome correction with pre- and probiotics<br>Sleep and stress management<br>Preventive strategies:

			- Bone health program (calcium, vitamin D, weight-bearing exercises)<br><br>- Cardiovascular health optimization<br><br>- Cancer screening and risk reduction strategies
Men's Hormonal Health and Longevity Program	Targets men aged 40 and older, focusing on those experiencing symptoms of hormonal imbalance or age-related health concerns.	Comprehensive hormone panel (blood test): testosterone (total and free), estradiol, DHEA, cortisol, thyroid hormones (TSH, T3, T4).<br><br>Cardiovascular risk assessment (lipid profile, blood pressure)<br><br>Bone density scan (DEXA)<br><br>Prostate-specific antigen (PSA) test<br><br>Body composition analysis<br><br>Metabolic panel (blood glucose, HbA1c)	Hormone Optimization:<br><br>- Testosterone replacement therapy (TRT) if indicated (topical gels, pellet implants, oral medications (newer formulations))<br><br>- Thyroid hormone optimization if needed<br><br>- DHEA supplementation if needed<br><br>Cardiovascular Health:<br><br>- Personalized exercise programs<br><br>- Nutritional counseling for heart health<br><br>- Medication management if needed (e.g., statins, blood pressure medications)<br><br>Weight Management:<br><br>- Customized diet plans<br><br>- Exercise regimens for fat loss and muscle gain<br><br>- Metabolic optimization<br><br>Cognitive Health:<br><br>- Brain health supplements<br><br>- Cognitive enhancement strategies<br><br>- Stress management techniques<br><br>Musculoskeletal Health:<br><br>- Resistance training programs

SUPPLEMENTARY DATA

			- Joint health supplements<br>- Osteoporosis prevention strategies
ResilientCare: Long COVID and Vaccine Sequelae Management Program	The program targets adults (18 years and older) who are experiencing persistent symptoms 12 weeks or more after acute COVID-19 infection, regardless of initial disease severity.	Interview and in-depth medical examination.<br><br>Instrumental diagnostics:<br>- Bio-Impedance measurement<br>- Ankle-brachial index (ABI)<br>- 24-hours ECG and blood pressure measurement<br>- Pulmonary function tests and chest imaging<br><br>Laboratory diagnostics:<br>- Immune status<br>- Metabolism<br>- Oxidative stress<br>- Antioxidant system<br>- Complete blood count<br>- Comprehensive metabolic panel<br>- Inflammatory markers (CRP, ESR, D-dimer, ferritin)<br>- Vitamin D, B12 levels<br>- Thyroid and adrenal function tests.<br><br>Neuropsychological evaluation for cognitive symptoms.<br><br>Sleep study if sleep disturbances are present.<br><br>Evaluation of mental health (depression/anxiety screening)	Pulmonary rehabilitation for respiratory symptoms<br>Graded exercise therapy and pacing strategies for post-exertional malaise<br>Cognitive rehabilitation for brain fog<br>Psychotherapy and behavioral modifications for mental health<br>Lifestyle modifications (sleep hygiene, stress reduction, etc.)<br>Nutritional support and supplements (Vit. D, Vit. C, omega-3s, etc.)
Immune system	The program aims to help the immune system cope with fatigue and everyday stress, as well as strengthen the body's defences. The program offers an assessment of the health status and gives medical recommendations so that the patient can change	Laboratory tests:<br>- Complete Blood Count (CBC)<br>- Comprehensive Metabolic Panel (CMP) to evaluate kidney and liver function, electrolyte and fluid balance, and blood sugar levels	- Nutritional supplements like zinc, vitamin C, vitamin D, elderberry and probiotics to support immune function<br>- Anti-inflammatory, nutrient-dense diet rich in colorful fruits and

	his/her lifestyle in the long term. This is a way to a more fulfilling life, where nutrition, physical activity and well-being will form a single unit for the immune system	- Vitamin D level to check for deficiency, which can impair immune function<br>- Immunoglobulins Panel to measure levels of IgG, IgA, and IgM antibodies<br>- Cellular Immune Status Panel using flow cytometry to assess T-cells, B-cells, and NK cells<br>- Micronutrient Testing to identify deficiencies in zinc, selenium, vitamin C, and B vitamins that can weaken immunity<br>- Comprehensive Stool Test to assess gut microbiome health, digestion, absorption, and intestinal permeability<br>- Salivary Cortisol Test to evaluate stress hormone levels that can suppress immune function<br>- Organic Acids Test to assess nutrient deficiencies, energy production, detoxification, and intestinal health	vegetables, healthy fats, fermented foods, and bone broth to nourish the immune system<br>- Regular moderate exercise, quality sleep, and stress management techniques to strengthen overall immune resilience
Weight control	The program focuses on restoring good physical shape and stabilizing metabolism with lasting results. The patient undergoes a health screening with metabolic rate measurement so that medical doctors get a comprehensive picture of his/her state of health. The program also contains an evaluation for the detection of possible malfunctions.	Comprehensive medical history and physical exam, including assessment of:<br>- Current weight, BMI, and waist circumference<br>- Weight history and prior weight loss attempts<br>- Dietary and exercise habits<br>- Medications and supplements<br>- Medical conditions and family history<br><br>Laboratory testing:<br>- Comprehensive metabolic panel (CMP) to assess glucose, electrolytes, kidney and liver function<br>- Lipid panel to evaluate cholesterol and triglyceride levels<br>- Thyroid stimulating hormone (TSH) to screen for thyroid dysfunction<br>- Hemoglobin A1c to assess diabetes risk<br>- Vitamin D level as low levels may hinder weight loss	-Personalized nutrition program<br>Customized fitness plan:<br>Combination of aerobic exercise and strength training<br>- Goal of 150-300 minutes per week of moderate intensity activity<br>- Supervision by certified fitness professionals<br>- Behavior modification:<br>Identification of triggers for overeating<br>- Stress management and relaxation techniques<br>- Cognitive behavioral therapy with licensed psychologist<br>- Support groups and group workshops<br><br>Medications and supplements (as appropriate):

© 2024. Mironov S. et al. Published online at http://www.aginganddisease.org/EN/10.14336/AD.2024.0328-1

		- Consider additional hormone testing as indicated (e.g. cortisol, sex hormones)<br><br>Body composition analysis:<br>- Bioelectrical impedance scale or DEXA scan to measure body fat percentage<br>- Waist circumference and waist-to-hip ratio<br>- Resting metabolic rate testing to determine caloric needs	- FDA-approved weight loss medications prescribed by physician<br>-Vitamin and mineral supplementation to prevent deficiencies<br>-Hormone replacement therapy to correct imbalances contributing to weight gain
Easy breath	The ecology of cities, constant stress and infections provoke the development of chronic diseases of the respiratory system. The cutting-edge diagnostic methods, combining the "Western" and "Eastern" approaches, allow to quickly create an updated map of the health.	Spirometry<br>CT scan<br>Arterial blood gas analysis: Measures oxygen and carbon dioxide levels in the blood to determine if the lungs are delivering enough oxygen.<br>Alpha-1 antitrypsin deficiency screening: Genetic testing for this condition, which increases COPD risk, may be done in younger patients or those with a family history.<br>Additional tests like electrocardiogram, echocardiogram, lung volume measurement, diffusing capacity, and oxygen level assessment	Inhaled Medications<br>Pulmonary Rehabilitation<br>A program combining exercise training, breathing techniques, nutritional counseling, and education

SUPPLEMENTARY DATA

The pleasure of movement	A sedentary lifestyle, a deficiency of microelements and amino acids, an unbalanced diet have an adverse effect on the condition of the bones, muscles and ligaments. The laboratory and instrumental diagnostics allow to create an updated map of the musculoskeletal system. A personal functional training plan has a positive effect on the physical activity and emotional state. The procedures included in the program eliminate the consequences of overload, restore muscle tone, improve the condition of the skin and ligaments.	Body composition analysis (DXA scan or bioelectrical impedance) to measure muscle mass, body fat percentage, and bone mineral density<br><br>Handgrip strength test to assess muscle strength<br><br>Physical performance tests like gait speed, chair stand test, and SPPB (Short Physical Performance Battery) to evaluate muscle function<br><br>Blood tests to check vitamin D levels,sex hormones, growth hormone, and inflammatory markers<br><br>Bone density scan (DXA) to screen for osteoporosis and fracture risk	Personalized resistance training program 2-3 times per week to build muscle mass and strength<br><br>Protein and amino acid supplementation to stimulate muscle protein synthesis, aiming for 1-1.5g protein per kg body weight daily<br><br>Vitamin D supplementation to maintain optimal levels for muscle and bone health<br><br>Hormone replacement therapy if indicated by blood tests - testosterone for men, estrogen for post-menopausal women<br><br>Anti-inflammatory nutrition plan rich in omega-3s, antioxidants, and plant foods to reduce inflammation<br><br>Bone-loading exercises like walking, jogging, dancing and weightlifting to increase bone mineral density<br><br>Fall prevention training including balance exercises and home safety assessment to reduce fracture risk
